# Lysine l‐Lactylation: Bridging Metabolism, Chromatin and Disease

**DOI:** 10.1111/cpr.70262

**Published:** 2026-07-12

**Authors:** Anoosha Malik, Muhammad Dilawar, Junguang Liao, Jie Zheng, Qitao Qian, Ming Xu, Sisi Lin, Xiaobo Zhu, Qiuwei Ge, Limin Jin, Guiqian Chen

**Affiliations:** ^1^ Department of Biopharmaceutics, Zhejiang Provincial Engineering Research Center of New Technologies and Applications for Targeted Therapy of Major Diseases, College of Life Science and Medicine Zhejiang Sci‐Tech University Hangzhou China; ^2^ Department of Orthopaedics The 903rd Hospital of the Joint Logistic Support Force Hangzhou China; ^3^ Department of Orthopaedic Surgery The First Affiliated Hospital, School of Medicine, Zhejiang University Hangzhou China; ^4^ Clinical Laboratory, Zhejiang Rehabilitation Medical Center Hangzhou Zhejiang China; ^5^ Clinical Laboratory, The Second Hospital of Jiaxing, The Second Affiliated Hospital of Jiaxing University Jiaxing China

**Keywords:** lactate, lysine lactylation, post‐translational modification (PTM), writer–eraser–reader proteins

## Abstract

Lysine L‐lactylation (K_L‐la_) is a newly identified metabolite‐derived post‐translational modification that directly bridges cellular metabolic states to chromatin regulation and protein function. Mounting evidence shows that K_L‐la_ has pivotal roles in transcription regulation and diverse cellular processes and is implicated in multiple pathophysiological conditions. This review comprehensively examines K_L‐la_ across both histone and non‐histone substrates in biology and disease. We first illustrate the historical development of K_L‐la_ and distinguish it from its isomers. We then delineate the enzymes regulating K_L‐la_, examine its crosstalk with other PTMs, and discuss its roles in cell signalling and other biological processes. Particular emphasis is placed on mechanisms through which K_L‐la_ contributes to various human diseases such as cancer, viral infections, neurodegenerative disorders, cardiovascular conditions, metabolic abnormalities and immune dysregulation. Finally, we provide an in‐depth analysis of emerging therapeutic strategies targeting K_L‐la_ and highlight future directions for translating mechanistic insights into clinical applications.

## Graphical abstract

Enzymatic regulation of K_L‐la_. The enzymatic regulation of K_L‐la_ involves two distinct pathways for L‐lactate accumulation: intracellular production through glycolysis‐derived pyruvate conversion by lactate dehydrogenase (LDH) or direct cellular uptake via monocarboxylate transporters (MCTs). These L‐lactate pools fuel two distinct lactylation pathways—lactyl‐CoA‐dependent and lactyl‐CoA‐independent (lactate/ATP‐dependent). In the lactyl‐CoA‐dependent mechanism, GTPSCS and ACSS2 convert L‐lactate into lactyl‐CoA, which serves as the donor for writers (KATs, including p300, CBP, KAT5, KAT7, KAT8 and KAT2A) to catalyse lactyl group transfer onto histone or non‐histone protein substrates. Beyond KATs, ATA1, ACAT2, ESCO2 and NAA10 are newly identified writers of the L‐lactyl co A dependent pathway. Alternatively, in the lactyl‐CoA‐independent pathway, AARSs catalyse lactylation via a two‐step reaction: first form Lac‐AMP intermediate through an ATP‐dependent manner, then transfer lactyl group to lysine residue with the release of AMP. K_L‐la_ is dynamically reversed by erasers (KDACs; SIRT1‐3,6 and CobB, HDAC1‐3 and 8), while readers such as Dux and TRIM33 interpret lactylation marks to regulate downstream signalling. This coordinated enzymatic system, encompassing writers (KATs, AARSs and others), erasers (KDACs) and readers (Dux, TRIM33), ensures precise spatiotemporal control of K_L‐la_ over protein function.

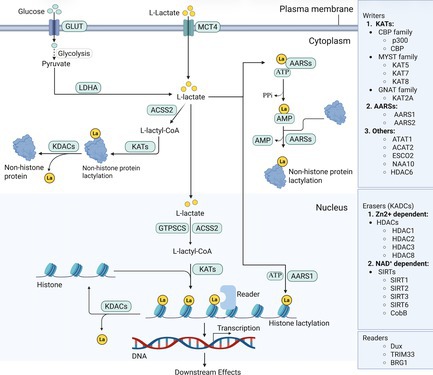

**Abbreviations**
AARSs: alanyl‐tRNA synthetases
ACSS2: acetyl‐CoA synthetase 2
GTPSCS: guanosine triphosphate‐specific succinyl‐CoA synthetase
KATs: lysine acetyltransferases
KDACs: lysine deacetylases

## 1 Introduction

The precise regulation of protein activity is essential to cellular homeostasis and physiology. Among the diverse mechanisms regulating protein function, post‐translational modifications (PTMs) have garnered considerable attention because they are implicated in almost every aspect of cellular, physiological and pathological processes by modulating protein stability, protein–protein interactions, intracellular trafficking and transcriptional activity [1, 2, 3, 4]. PTMs also serve as an important bridge between cellular metabolism and protein function regulation as many metabolites (e.g., acetoacetyl‐CoA, crotonyl‐CoA) serve directly as substrates for PTMs [2].

Lysine L‐lactylation (K_L‐la_) is a newly identified, metabolite‐derived PTM in which L‐lactate serves as a precursor for transfer of the L‐lactyl group to lysine residues of both histone and non‐histone proteins [5]. Mounting evidence implicates K_L‐la_ in a wide range of biological processes including transcriptional regulation, cell fate decisions, embryonic development and DNA repair [5, 6, 7, 8]. Furthermore, K_L‐la_ associates with various human diseases. For example, L‐lactylation of histone 3 at lysine 18 (H3K18la) is implicated in cancer progression, including colorectal cancer [9], bladder cancer [10] and ocular melanoma [11]. It also modulates immune response [12], liver fibrosis [13] and myocardial infarction (MI) [14]. K_L‐la_ of non‐histone proteins such as adenylate kinase 2 (AK2) [15] and cyclin E2 (CCNE2) [16] promotes hepatocellular carcinoma (HCC), while lactylated α‐myosin heavy chain (α‐MHC) associates with heart failure [17]. In sum, K_L‐la_ plays an indispensable role in various biological and pathological processes.

K_L‐la_ exhibits extensive crosstalk with other PTMs, forming integrated regulatory networks. Despite sharing lysine residues and metabolic origins with acetylation (K_ac_), K_L‐la_ exhibits distinct structural features, metabolic dynamics and site preferences, leading to both competitive and cooperative interactions [18, 19, 20]. K_L‐la_ also interfaces with ubiquitination in a protein‐ and context‐dependent manner, thereby fine‐tuning protein stability, signalling and transcriptional regulation [21, 22, 23]. Additionally, K_L‐la_ modulates various signalling pathways orchestrating a wide array of cellular functions [24, 25].

By synthesizing the latest advances, this review provides a comprehensive examination of K_L‐la_ across both histone and non‐histone substrates in biology and disease. We begin by illustrating the historical development of K_L‐la_ and distinguishing it from its isomers. We then delineate the enzymes regulating K_L‐la_ and examine its crosstalk with other PTMs. We further discuss the emerging roles of K_L‐la_ in diverse cell signalling and many other biological processes. Particular emphasis is placed on pathophysiological functions and underlying mechanisms through which K_L‐la_ contributes to various human diseases. Finally, we provide an in‐depth analysis of emerging therapeutic strategies targeting K_L‐la_ and propose a translational framework for future clinical investigations.

### 1.1 History of K_L‐la_ Research

**FIGURE 1:**
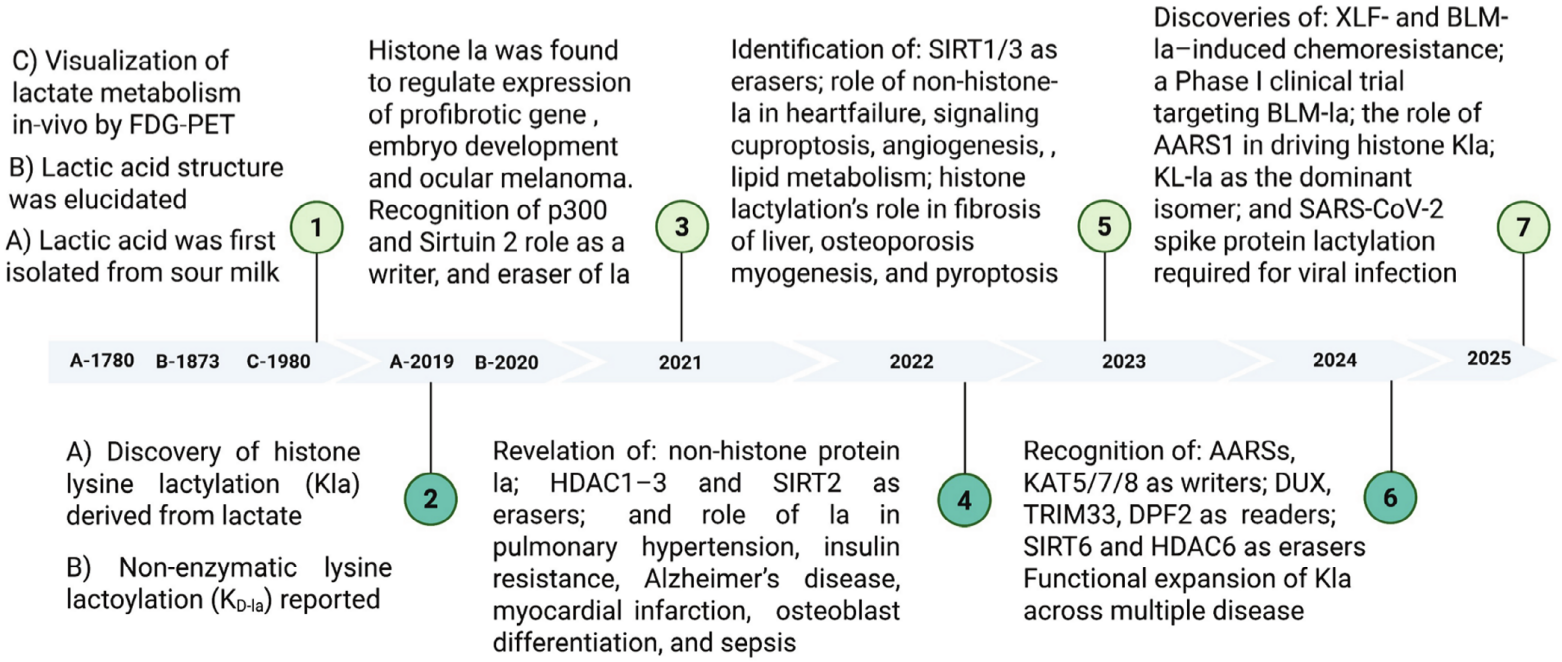
Research history of lysine L‐lactylation. AARSs, alanyl‐tRNA synthetases; DPF2, double PHD fingers 2; la, L‐lactylation.

Lactate (C_3_H_5_O_3_^−^) is an ionized form of lactic acid (C_3_H_6_O_3_), produced upon lactic acid dissociation in water [26, 27]. Lactic acid was first isolated from sour milk by Swedish chemist Carl Wilhelm Scheele in 1780. In 1808, Jöns Jacob Berzelius found that lactic acid is also generated by muscle tissues during exercise, and in 1873 Johannes Wislicenus elucidated its structure [28]. Since then, lactate has merely been regarded as a metabolic waste product of glycolysis for more than a century [28, 29]. This view gradually changed as accumulating evidence showed that lactate serves as a crucial metabolic fuel for skeletal muscle, heart, brain and malignant cells, and contributes to cell fate determination and intercellular signalling [30, 31, 32, 33, 34, 35]. This evidence positioned lactate as a bioactive metabolite and signalling molecule rather than merely a by‐product of energy metabolism (Figure 1).

A major breakthrough occurred in 2019 when Zhang et al. identified K_L‐la_ on histones through sophisticated mass spectrometry and stable isotope metabolic labelling approaches [5]. This discovery provided the first direct molecular link between lactate metabolism and epigenetic regulation, revealing lactate as a metabolite capable of influencing gene expression through covalent protein modification. Subsequent investigations in 2022 expanded K_L‐la_ beyond histones and demonstrated its widespread occurrence on many non‐histone proteins, largely broadening its potential biological functions [36, 37, 38]. In parallel, identification of specific writers, erasers and readers revealed that K_L_‐la is dynamically regulated and reversible PTM [39, 40, 41, 42]. These findings established K_L_‐la as a tightly controlled PTM, stimulating far‐reaching investigations into its physiological and pathological roles (Figure 1).

More recent studies have revealed diverse roles for K_L‐la_ in multiple physiological and pathological processes, including transcriptional regulation, DNA damage repair, tumour progression, immune responses and host–pathogen interactions [3, 43, 44]. Together, these breakthroughs have advanced our understanding of K_L‐la_, positioning it as a key regulatory mechanism linking lactate metabolism to diverse biological processes (Figure 1).

### 1.2 Distinctions Between K_L‐la_ and Its Isomers

**FIGURE 2:**
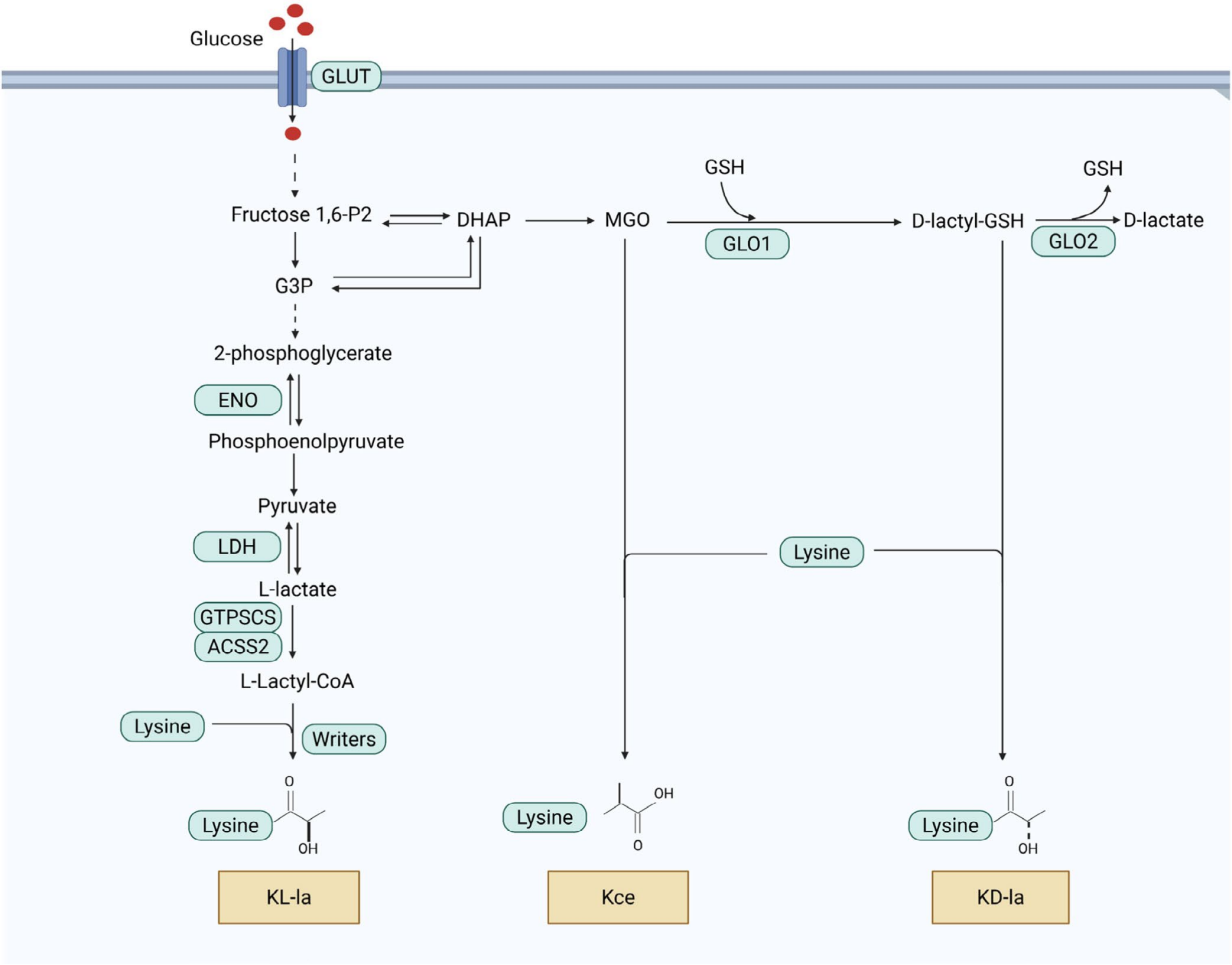
Distinctions between K_L‐la_ and its Isomers. Lysine L‐lactylation (K_L‐la_) is enzymatically formed from L‐lactate. N‐ε‐(carboxyethyl)‐lysine (K_ce_) and lysine d‐lactylation (K_d‐la_) are non‐enzymatically derived from S‐methylglyoxal (MGO) and D‐lactoylglutathione (D‐LGSH), respectively. MGO is formed by spontaneous fragmentation of two glycolysis intermediates, dihydroxyacetonephosphate (DHAP) and glyceraldehyde‐3‐phosphate (G3P). The glyoxalase system, encompassing glyoxalase 1 (GLO1) and glyoxalase 2 (GLO2), converts MGO to D‐LGSH and subsequently to d‐lactate.

Lysine lactylation involves the covalent addition of L‐lactyl groups to ε‐amino lysine residues on target proteins. Stereochemical analyses have revealed three structurally distinct isomers of lysine lactylation: K_L‐la_; lysine ε‐N‐carboxyethylation (K_ce_); and lysine d‐lactylation (K_d‐la_). Among these, K_L‐la_ represents the sole glycolysis‐ and hypoxia‐responsive lactylation that is enzymatically regulated and derived from its precursor, _L_‐lactate [20, 45]. In contrast, K_ce_ and K_d‐la_ are formed non‐enzymatically via reaction of the lysine side chain's amine group with glycolytic byproducts, methylglyoxal (MGO) and S‐D‐lactoylglutathione (D‐LGSH), respectively [46] (Figure 2). Notably, physiological concentrations of MGO (< 10 μM intracellular) and D‐LGSH (~20 μM in plasma) [47] are substantially lower than L‐lactate (1.5–3.0 mM) [48], providing a plausible explanation for the lower abundance of K_ce_ and K_d‐la_ in contrast to K_L‐la_. However, the relative quantitative and physiological contributions of enzymatic K_L‐la_ and non‐enzymatic lactoylation to the cellular lactylome remain to be determined. In addition, K_L‐la_ has been extensively studied across diverse biological systems [3, 6, 44, 49, 50, 51, 52, 53, 54, 55, 56, 57, 58, 59, 60, 61, 62, 63, 64, 65], whereas the biological functions of Kce and K_d_‐_la_ remain largely unexplored.

### 1.3 Enzymes Regulating K_L‐la_

**FIGURE 3:**
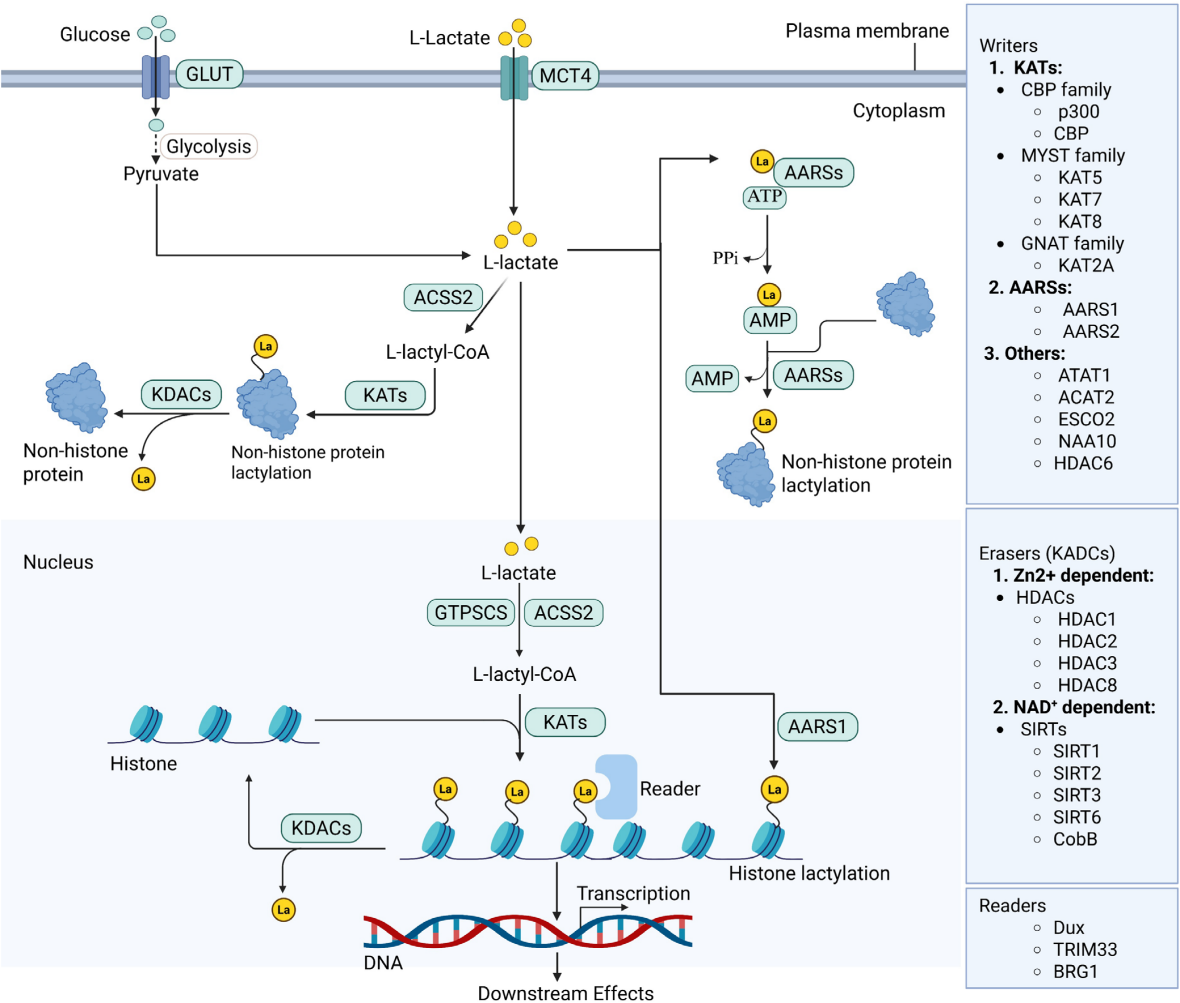
Enzymatic regulation of K_L‐la_. The enzymatic regulation of K_L‐la_ involves two distinct pathways for L‐lactate accumulation: Intracellular production through glycolysis‐derived pyruvate conversion by lactate dehydrogenase (LDH) or direct cellular uptake via monocarboxylate transporters (MCTs). These L‐lactate pools fuel two distinct lactylation pathways—lactyl‐CoA‐dependent and lactyl‐CoA‐independent (lactate/ATP‐dependent). In the lactyl‐CoA‐dependent mechanism, GTPSCS and ACSS2 convert L‐lactate into lactyl‐CoA, which serves as the donor for writers (KATs, including p300, CBP, KAT5, KAT7, KAT8 and KAT2A) to catalyse lactyl group transfer onto histone or non‐histone protein substrates. Beyond KATs, ATA1, ACAT2, ESCO2 and NAA10 are newly identified writers of L‐lactyl co A dependent pathway. Alternatively, in the lactyl‐CoA‐independent pathway, AARSs catalyse lactylation via two‐step reaction: First form Lac‐AMP intermediate through an ATP‐dependent manner, then transfer lactyl group to lysine residue with release of AMP. K_L‐la_ is dynamically reversed by erasers (KDACs; SIRT1‐3,6 and CobB, HDAC1‐3 and 8), while readers such as Dux and TRIM33 interpret lactylation marks to regulate downstream signalling. This coordinated enzymatic system, encompassing writers (KATs, AARSs and others), erasers (KDACs) and readers (Dux, TRIM33), ensures precise spatiotemporal control of K_L‐la_ over protein function. Abbreviations: AARSs, Alanyl‐tRNA synthetases; ACSS2, acetyl‐CoA synthetase 2; GTPSCS, guanosine triphosphate‐specific succinyl‐CoA synthetase; KATs, lysine acetyltransferases; KDACs, lysine deacetylases.

K_L‐la_ is dynamically regulated by lactyltransferases (writers), delactylases (erasers), whereas L‐lactyl‐lysine binding proteins (readers) recognize K_L‐la_ and mediate its downstream effects (Figure 3).

#### 1.3.1 Lactyltransferases Catalysing the Addition of K_L‐la_

The lactyltransferases responsible for addition of K_L‐la_ can be categorized into two groups: lysine acyltransferases (KATs) and alanyl‐tRNA synthetases (AARSs). The K_L‐la_ is driven by cellular L‐lactate availability, which originates from two primary sources: monocarboxylate transporter (MCT)‐mediated transmembrane transport of extracellular L‐lactate and intracellular glycolysis, where glucose‐derived pyruvate is reduced to L‐lactate by lactate dehydrogenase (LDH). Intracellular L‐lactate can subsequently drive two K_L‐la_ pathways—L‐lactyl‐CoA‐dependent and L‐lactyl‐CoA‐independent (Figure 3).

##### 1.3.1.1 KAT‐Mediated Lactyl‐CoA‐Dependent K_L‐la_

In the lactyl‐CoA‐dependent pathway, GTP‐dependent succinyl‐CoA synthetase (GTPSCS) and acetyl‐CoA synthetase 2 (ACSS2) convert L‐lactate into lactyl‐CoA, which serves as the lactyl donor for KATs to catalyse transfer of the lactyl group onto lysine residues [20, 60, 66, 67] (Figure 3). Although KATs were traditionally identified as acetyltransferases, structural features of their catalytic pocket allow them to accommodate multiple acyl‐CoAs including l‐lactyl‐CoA, thus enabling K_L‐la_. KATs comprise three major families: (1) the CBP family encompassing p300 and CBP; (2) MYST family including KAT5 (also known as TIP60), KAT7 (HBO1) and KAT8 (MOF); and (3) GNAT family comprising KAT2A (GCN‐5), KAT2B and HAT1 [68].

The p300 catalyses K_L‐la_ of both histone [5, 12, 39, 69, 70], and non‐histone substrates [71, 72] in diverse cell types. MYST family demonstrates robust K_L‐la_ activity, with KAT7 catalysing H3K9la [73], KAT5 lactylating Nijmegen breakage syndrome (NSB1) at K388 (NSB1‐K388la) [74], and KAT8 targeting elongation factor 1 alpha (eEF1A2) at K408 [42]. The GNAT family member KAT2A strongly lactylates H3 at K18 and K14 [14, 66] and extracellular signal–regulated kinase (ERK) at K231 [24].

It is notable that, beyond KATs, several non‐canonical acetyltransferases including ATAT1, ACAT2, ESCO2 and NAA10 have recently been identified as lactyltransferases, catalysing the L‐lactylation of N‐acetyltransferase 10 (NAT10) [75], glutamate‐cysteine ligase modifier subunit (GCLM; K34) [76], AlkB homologue 5 (ALKBH5; K284) [77] and NOP2/Sun RNA methyltransferase family member 2 (NSUN2; K508), respectively [58, 75].

Although progressive research has reported a substantial enzymatic role of canonical KATs and non‐canonical acetyltransferases in regulating K_L‐la_, the structural and mechanistic basis of this modification is yet largely unresolved. Future exploration should focus on deciphering the catalytic architecture and substrate recognition mechanisms that facilitate these enzymes to accommodate lactyl‐CoA. Equally important is the quantitative assessment of their catalytic kinetics, which would exhibit the extent to which individual acetyltransferases contribute to whole lactylation flux. Additionally, because enzyme activity and subcellular localization can differ with cellular context and physiological state, investigations should integrate cell type–specific and pathophysiological condition–dependent analyses to understand well the dynamic regulation of lactylation in vivo.

##### 1.3.1.2 AARS‐Mediated Lactyl‐CoA‐Independent K_L‐la_

In lactyl‐CoA‐independent mechanism, L‐lactate is activated by AARS1 and AARS2 in an ATP‐dependent manner to form L‐lactyl‐AMP. The lactyl group is then transferred from L‐lactyl‐AMP to the lysine residue, thus bypassing the need for L‐lactyl‐CoA and KAT‐like lactyltransferases [18, 78, 79, 80] (Figure 3).

AARS1 regulates histone lactylation including H3K18la [78, 79], and lactylates multiple non‐histone proteins: tumour protein p53 (p53; K120/K139) [18]; nudix hydrolase 21 (NUDT21; K23) [81]; Bloom syndrome protein (BLM; K24) [3]; signal transducer and activator of transcription 1 (STAT1; K193) [79]; Yes‐associated protein (YAP; K90); and TEA domain 1 (TEAD1; K108) [78]. AARS2 primarily targets non‐histone substrates including cyclic GMP–AMP synthase (cGAS), pyruvate dehydrogenase E1 subunit alpha1 (PDHA1; K336) and carnitine palmitoyltransferase 2 (CPT2; K457/8) [82], whereas its role in histone K_L‐la_ remains to be explored.

Kinetically, AARS1 has lower preference for L‐lactate (*K*_m_ = 36 mM) as compared to its canonical substrate L‐alanine (*K*_m_ = 80 μM), and it has lower binding affinity to L‐lactate (*K*_d_ = 35 μM) than L‐alanine (*K*_d_ = 0.45 μM) [18, 80]. Considering that the intracellular L‐alanine levels (~0.5 mM) are comparable with physiological L‐lactate levels (1.5–3.0 mM) [48, 83], L‐alanine is likely the preferred substrate for AARS1, thus it can inhibit L‐lactyl‐AMP formation and subsequently K_L‐la_. However, in cancer tissue, elevated lactate concentrations (10–40 mM) [84], could approach the *K*_m_ of AARS1 for lactate. Therefore, although the *k*_cat_/*K*_m_ towards lactate is lower than that towards L‐alanine, higher intratumoral lactate concentrations may promote the production of L‐lac‐AMP and K_L‐la_. Notably, the cytosolic isoform AARS2 demonstrates lower *K*_m_ (5 mM) and more catalytic efficiency [80].

Given that AARSs have dual functions in K_L‐la_ and canonical aminoacylation, future research should use separation‐of‐function strategies to determine whether the phenotypic consequences of AARS1/2 deficiency result from reduced K_L‐la_ or from impaired alanyl‐tRNA generation and protein synthesis.

##### 1.3.1.3 Delactylases Removing K_L‐la_

The removal of lactyl moiety from lysine residues is mediated by two major classes of lysine deacetylases (KDACs): the zinc‐dependent histone deacetylases (HDAC1‐3 and 8) and NAD^+^‐dependent sirtuins (SIRT1‐3,6 and CobB) [85, 86, 87] (Figure 3).

Multiple studies have shown that HDAC1‐3 remove K_L‐la_ on diverse substrates across different cell lines [40, 69, 74, 88, 89, 90]. Additionally, multiprotein complexes encompassing HDAC1 such as CoREST, MiDAC and RERE also exhibit delactylase activity [91]. SIRT1 exhibits strong delactylase activity towards various substrates including α‐myosin heavy chain (α‐MHC; K1897) [17], alpha‐enolase (ENO1; K22) [92], YAP (K90), TEAD (K108) [78], M2 splice isoform of pyruvate kinase (PKM2; K207) [92], and meiotic recombination 11 (MRE11; K673) [67]. SIRT2 has also been shown to possess strong delactylase activity [93, 94]. SIRT3 targets both lactylated histone and non‐histone proteins such as H4K16 [95], cyclin E2‐K348 [96], PDHA1 (K336), CPT2 (K457/8) [82] and aldehyde dehydrogenase 2 (ALDH2; K52) [63]. More recently, SIRT6 has been observed as an efficient delactylase removing H3K9la on in vitro‐reconstituted nucleosomes [97, 98]. Whether other HDAC and sirtuin family members, including HDAC10 and HDAC11, also exhibit delactylase activity remains to be determined.

It is plausible that delactylases exert context‐dependent roles, as their distinct subcellular localization, expression levels and the availability of cofactor and precursors can influence their enzymatic activities [94]. Future research should focus to fill the gaps that remain regarding: (1) proteome‐wide identification of delactylase substrates, (2) structural determinants of lactyl‐group specificity and (3) mechanisms coordinating the spatial and temporal regulation of K_L‐la_ dynamics. Systematic characterization of delactylase networks will be essential for understanding their role in metabolic‐epigenetic crosstalk and developing targeted interventions for diseases.

##### 1.3.1.4 Readers of K_L‐la_

The recognition and interpretation of K_L‐la_ marks by specialized ‘reader’ proteins represents a critical yet understudied aspect of lactylation signalling. PHD finger domain‐containing proteins (BPTF, DPF2 and ING), bromodomain‐containing proteins (BRD2, BRD4 and TRIM33β) and YEATS domain proteins (YEATS2, AF9, ENL and GAS41) are known as traditional readers of acetylation [99, 100]. Owing to structural homology among acetyl and lactyl groups, some of these acetylation readers may also function as readers of K_L‐la_. Indeed, BRG1, a bromodomain‐encompassing chromatin remodeler, and TRIM33β have been recognized as readers of H3K18la [41, 101]. Likewise, DPF2 serves as a reader of H3K14la [102] (Figure 3).

Future studies should investigate the structural basis of lactyl‐mark recognition by different reader domains; potential crosstalk with other acylation reader proteins; and functional consequences of reader‐K_L‐la_ interactions in gene regulatory networks. Moreover, quantitative biophysical approaches should be employed to characterize binding affinities, high‐resolution structural biology to define interaction interfaces, and genome‐wide mapping to elucidate reader‐dependent transcriptional outcomes. As the emerging paradigm positions K_L‐la_ readers as key integrators of metabolic state and chromatin regulation, with particular relevance for physiological and pathological processes including cellular reprogramming [101] and tumorigenesis [102]. Systematic investigation of these recognition systems will be crucial for understanding how K_L‐la_ transduces metabolic information into specific biological responses.

### 1.4 Enzymatic Versus Non‐Enzymatic K_L‐la_

Although existing literature strongly suggests that K_L‐la_ is mainly an enzyme‐catalysed modification, as supported by several evidences: First, in vitro assays have demonstrated that multiple lactyltransferases such as KATs and AARSs can directly mediate K_L‐la_ using L‐lactyl‐CoA or L‐lactate respectively [5, 14, 42, 73, 74, 78, 79]. Second, inhibition of these lactyltransferases decreases K_L‐la_ levels across different cell lines, endorsing their intracellular activities [5, 20, 38, 42, 67, 73, 74, 103, 104]. However, K_L‐la_ may not be exclusively enzyme‐directed in all systems, as recent work in Salmonella demonstrates non‐enzymatic formation of K_L‐la_ derived from L‐lactyl‐CoA [105]. Together, these findings support a model in which K_L‐la_ is predominantly enzyme‐controlled in mammalian cells, while non‐enzymatic chemistry may contribute to specific organisms or metabolic states.

### 1.5 K_L‐la_ Crosstalk With Other PTMs

PTMs exhibit complex crosstalk, whereby one modification can influence another through competitive, cooperative, hierarchical, or context‐dependent mechanisms. Emerging evidence indicates that K_L‐la_ coordinate extensively with other PTMs, including acetylation and ubiquitination through interactions.

Competitive crosstalk occurs when K_L‐la_ and other PTMs target the same lysine residue. For example, L‐lactylation of p53 at K120 and K139 prevents acetylation at these sites, impairing p53 DNA‐binding affinity and liquid–liquid phase separation [18]. Similarly, p300 induces both lactylation and acetylation of PKM2 at K433 [71, 106]. Mannose‐mediated suppression of lactate formation shifts PKM2 modification from lactylation to acetylation, facilitating PKM2 nuclear localization and triggering nuclear factor kappa‐light‐chain‐enhancer of activated B cells (NF‐κB)‐dependent pyroptosis in bladder cancer [71]. Another example is α‐tubulin K40, a residue that can undergo either L‐lactylation or acetylation. L‐lactylation of K40, catalysed primarily by HDAC6 under high‐lactate conditions, promotes microtubule dynamics, neurite outgrowth and axonal regeneration, whereas acetylation of the same residue marks stable microtubules and supports structural integrity [94]. Together, this evidence indicates that site‐specific competition between K_L‐la_ and acetylation can function as a molecular switch that modulates protein function and downstream outcomes.

Cooperative and hierarchical crosstalk occurred when K_L‐la_ regulates the deposition or function of other PTMs. Histone K_L‐la_ upregulates the m6A reader YTH domain‐containing protein 1 (YTHDC1), which in turn recruits p300 to enhance histone acetylation at lipogenic gene promoters, thereby driving metabolic reprogramming and HCC progression [65]. Likewise, transforming growth factor beta 1 (TGFβ1)‐induced L‐lactylation of USP2 at K447 enhances its deubiquitinase activity. Recently Tu et al. revealed a multilayered PTM network, in which K_L‐la_ upregulates ubiquitin‐specific protease 4 (USP4), while acetylation of Annexin A2 (ANXA2) promotes its binding with USP4. USP4‐mediated deubiquitination stabilizes ANXA2 and enables downstream bone marrow tyrosine kinase gene in chromosome X (BMX)–signal transducer and activator of transcription 3 (STAT3) axis, thereby promoting glioma stem cell maintenance and radioresistance [56]. Collectively, these findings suggest that K_L‐la_ can act upstream of other PTM pathways and exert hierarchical regulatory effects.

K_L‐la_ mediated regulation of ubiquitination is highly substrate dependent. For example, inhibition of BLM lactylation promotes its ubiquitination and degradation [3], whereas K_L‐la_ of NSUN2 suppresses ubiquitination, thereby stabilizing the protein and enhancing 5‐methylcytosine (m5C) RNA methylation of CDCP1 and STC1 transcripts in pancreatic ductal adenocarcinoma (PDAC) [22]. In contrast, hypoxia‐induced K_L‐la_ of axis inhibition protein 1 (Axin1) promotes its ubiquitination [21]. These studies suggest that K_L‐la_ can either stabilize or destabilize proteins through modulation of ubiquitination pathways, with the outcome determined by the specific substrate and cellular context.

Collectively, current evidence highlights that crosstalk between K_L‐la_ and other PTMs can be competitive, cooperative, hierarchical, or substrate dependent. Future research should systematically delineate the regulatory relationship between K_L‐la_ and acetylation, specifically the mechanisms dictating modification selection at shared lysine residues. Although K_L‐la_ and acetylation often take place at similar sites and are linked with identical biological processes, it remains unclear whether they demonstrate functional redundancy or trigger different transcriptional programs by recruiting different readers. These two PTMs may occur at different time points, in distinct cell types, or under different pathological conditions, underscoring the need for high‐spatiotemporal‐resolution techniques for dynamic tracking of chromatin‐associated changes.

### 1.6 K_L‐la_ in Cell Signalling

**FIGURE 4:**
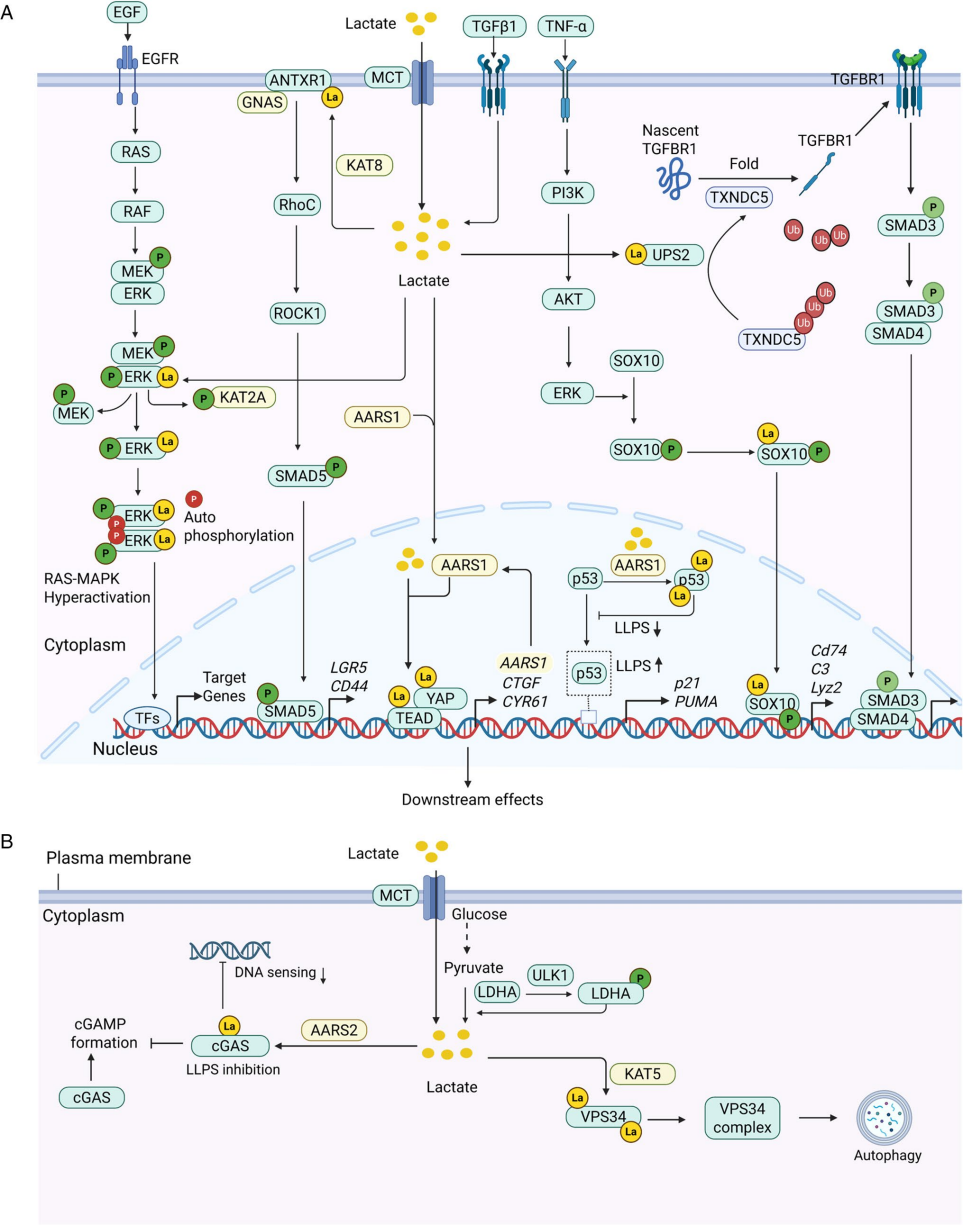
(A) Lysine L‐lactylation in cell signalling. K_L‐la_ modulates multiple signalling pathways influencing multiple cellular processes. In MAPK signalling, L‐lactylation of ERK promotes its activation through a KAT2A‐dependent positive feedback loop. K_L‐la_ stabilizes ANTXR1 to trigger RhoC/ROCK1/SMAD5 signalling and subsequently induces cancer stemness and therapeutic resistance. K_L‐la_ activates oncogenic programs by enhancing YAP–TEAD1 activity through AARS1. K_L‐la_ of p53 impairs its DNA binding, disrupts liquid–liquid phase separation (LLPS) and reduces its transcriptional activity towards downstream genes. PI3K–AKT–dependent Sox10 lactylation activates transcription of vascular inflammation‐related genes. TGFβ1‐induced K_L‐la_ of USP2 stabilizes TGFβR1 via TXNDC5 deubiquitination, reinforcing profibrotic responses. (B) Lysine L‐lactylation in cell signalling. Under stress, MCT‐dependent lactate influx facilitates AARS2‐catalysed K_L‐la_ of innate immune DNA sensor cGAS to disrupt its LLPS, thereby inhibiting cyclic GMP–AMP (cGAMP) formation and suppressing immune activity. Under nutrient deprivation, LDHA‐driven lactate accumulation supports KAT5‐mediated K_L‐la_ of VPS34, enhancing its interaction with autophagy proteins to promote autophagic flux.

Precise regulation of various cell signalling pathways is essential for regulation of human development and tissue homeostasis [107, 108, 109, 110, 111, 112, 113]. K_L‐la_ is increasingly recognized as a critical modulator of various signalling pathways (Figure 4A,B). In the following, we delineate how this modification regulates cellular processes by influencing cell signalling:

K_L‐la_ directly activates mitogen‐activated protein kinase (MAPK) signalling pathway. K_L‐la_ of ERK disrupts its association with MEK, facilitating ERK dimerization and promoting its activation. Activated ERK subsequently phosphorylates GCN5, which augments the enzyme's capacity to catalyse ERK lactylation, thereby forming a positive feedback circuit that promotes lactate‐driven tumour progression [24]. Interestingly, targeted inhibition of ERK L‐lactylation suppresses tumour growth in KRAS‐mutant models [24]. K_L‐la_ also orchestrates TGFβ pathway in organ fibrosis [25]. Specifically, TGFβ1 induced‐K_L‐la_ of USP2 facilitates deubiquitination of thioredoxin domain containing 5 (TXNDC5), an endoplasmic reticulum protein that stabilizes TGFβ receptor 1 (TGFβR1) to activate TGFβ signalling [25].

In addition, K_L‐la_ modulates signalling pathways that orchestrate cell proliferation. Intracellular lactate accumulation promotes nuclear translocation of AARS1, where it mediates K_L‐la_ and activation of the YAP‐TEAD1 complex, inducing downstream target gene expression and promoting gastric cancer cell proliferation [78]. Interestingly, AARS1 itself serves as a downstream target gene for YAP‐TEAD1, establishing a positive‐feedback loop [78]. Under hypoxia, K_L‐la_ promotes colorectal cancer (CRC) cell proliferation and stemness by modulating the activity of the Wnt signalling pathway [114]. K_L‐la_ also mediates tumour–stroma metabolic crosstalk to activate oncogenic signalling in CRC. L‐lactate derived from glycolytic cancer‐associated fibroblasts (CAFs) induces K_L‐la_ of anthrax toxin receptor 1 (ANTXR1) and activates the downstream RhoC/ROCK1/SMAD5 signalling cascade, leading to cancer stemness and chemoresistance [115]. Moreover, K_L‐la_ impairs tumour suppressor signalling, as L‐lactylation of p53 prevents its liquid–liquid phase separation (LLPS) and DNA‐binding, ultimately suppressing its transcriptional function and increasing oncogenesis [18].

K_L‐la_ also influences innate immune and inflammatory signalling cascades. Particularly, in response to stress, L‐lactate production is enhanced, and L‐lactate enters the cells through MCT1. AARS2 senses intracellular L‐lactate deposition and lactylates cyclic GMP–AMP synthase (cGAS), impairing its LLPS and DNA sensing, thereby suppressing cyclic GMP–AMP synthesis and dampening innate immune function [116]. Interestingly, MCT1 blockage prevents K_L‐la_ of cGAS in stressed mice, restoring innate immune sensing and counteracting viral DNA replication [116]. In vascular smooth muscle cells (VSMCs), inflammatory stimulation by tumour necrosis factor α (TNF‐α) activates the PI3K‐AKT signalling pathway, leading to phosphorylation of the transcription factor SOX10. This phosphorylation promotes K_L‐la_ of SOX10. Lactylated SOX10 exhibits altered transcriptional activity, inducing the expression of genes that facilitate VSMC trans differentiation into macrophage‐like cells and triggering pyroptotic cell death [89].

### 1.7 Biological Functions of K_L‐la_

K_L‐la_ serves as a fundamental molecular interface that couples cellular metabolic states with both epigenetic and non‐epigenetic regulatory circuits, orchestrating a wide spectrum of biological processes.

#### 1.7.1 Cell Fate Regulation and Reprogramming

**FIGURE 5:**
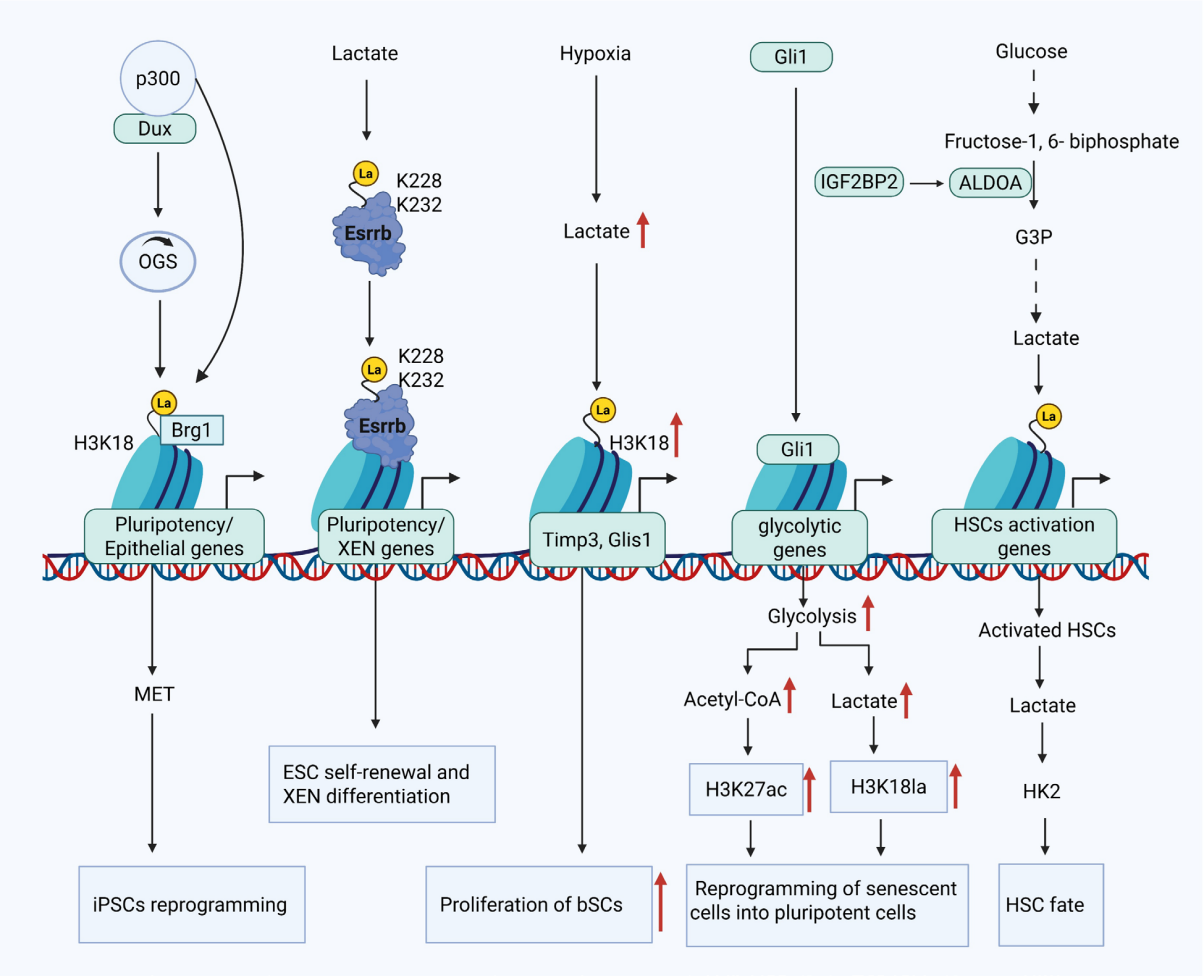
Lactylation‐mediated regulation of cell fate determination and reprogramming. Lactylation serves as a metabolic‐epigenetic nexus coordinating cellular plasticity across diverse biological contexts. In induced pluripotent stem cell (iPSC) generation, the transcription factor Dux initiates metabolic reprogramming by upregulating oxidative glycolysis (OGS)‐derived lactate production, which facilitates p300‐mediated H3K18 lactylation (H3K18la) deposition. This modification recruits the chromatin remodeler Brg1 to establish a metabolism‐lactylation‐MET (mesenchymal‐epithelial transition) axis, significantly promoting reprogramming efficiency. In embryonic stem cells (ESCs), lactylation of the pluripotency factor Esrrb at K228/K232 enhances its DNA‐binding capacity, simultaneously reinforcing self‐renewal while priming extraembryonic endoderm (XEN) lineage commitment—demonstrating lactylation's dual role in maintaining stemness and directing differentiation. Hypoxia induced H3K18la promotes proliferation of buffalo spermatogonial cells (bSCs). During somatic cell reprogramming, the pioneer factor Glis1 triggers a metabolic switch by opening chromatin at glycolytic gene loci, elevating intracellular acetyl‐CoA and lactate levels to co‐ordinately drive H3K27 acetylation (H3K27ac) and H3K18la deposition, thereby licensing chromatin accessibility and overcoming senescence barriers. In hepatic stellate cells (HSCs), the m6A reader IGF2BP2 mechanistically links glycolytic activation (via ALDOA modulation) to histone lactylation, establishing a self‐reinforcing activation loop further amplified by lactate‐induced hexokinase 2 (HK2) expression. Genetic ablation or pharmacological inhibition of this lactate‐HK2‐lactylation axis abolishes HSC activation, highlighting its essential role in cell fate determination. Abbreviations: MET (mesenchymal‐epithelial transition), OGS (oxidative glycolysis system), XEN (extraembryonic endoderm), ALDOA (aldolase A).

K_L‐la_ functions as a central metabolic‐epigenetic nexus, dynamically coupling cellular metabolism with gene regulatory programs to determine cell fate (Figure 5). For example, H3K18la facilitates induced pluripotent stem cells (iPSCs) reprogramming by regulating mesenchymal‐epithelial transition (MET) related genes [101]. Similarly, K_L‐la_ of the core pluripotency factor Esrrb enhances its DNA‐binding affinity, reinforcing ESC self‐renewal and directing extraembryonic endoderm stem (XEN) lineage specification [7]. Hypoxia activates glycolysis and enhances H3K18la and expression of its target genes including *Timp3* and *Glis1*, thereby triggering the proliferation of buffalo spermatogonial cells (bSCs) [6]. Moreover, Glis1 activates glycolytic genes and represses somatic loci, leading to elevated lactate levels which in turn enhance H3K18la at pluripotency genes and establish an open chromatin state that promotes reprogramming efficiency [117]. The m6A reader insulin‐like growth factor 2 mRNA‐binding protein 2 (IGF2BP2) regulates glycolysis, induces histone K_L‐la_ and promotes hepatic stellate cells (HSCs) activation [118], that induces hexokinase 2 (HK2) expression, determining HSC fate [13]. Accordingly, another study reported that K_L‐la_ inhibition or HK2 deletion represses HSC activation [13]. This evidence establishes K_L‐la_ as a versatile metabolic rheostat, transducing nutrient cues into chromatin‐based fate decisions through both histone and transcription factor modification.

#### 1.7.2 Embryonic Development and Regulation

K_L‐la_ acts as an important regulator of early embryogenesis, where glycolysis‐generated L‐lactate directly modifies chromatin to regulate developmental programming. Spatial mapping exhibited pronounced K_L‐la_ enrichment in metabolically active embryonic tissues such as neural crest cells and mesodermal precursors [119], while H3K18la dynamically regulates endometrial receptivity and successful implantation in vivo [120]. Mechanistically, H3K18la exerts dual functions in maintaining glutathione‐mediated redox balance essential for embryo viability [120] and activating cleavage‐stage genes like Zscan4 through enhanced transcriptional elongation [121]. Moreover, zygotic gene activation (ZGA) is required during early mammalian preimplantation development [122]. L‐Lactate regulates ZGA through H3K18la in mice and human embryos [8]. Strikingly, the lactylation‐depleted condition—whether through hypoxia [123], lactate deprivation [124] or LDHA inhibition [123]—consistently compromises preimplantation development, establishing H3K18la (rather than H3K27ac) as the dominant lactate‐mediated epigenetic mark driving embryogenesis [124].

Beyond preimplantation, histone K_L‐la_ integrates metabolic and signalling cues during tissue morphogenesis. In cranial neural crest cells (CNCCs) [125], bone morphogenetic protein (BMP) signalling regulates glycolytic‐induced L‐lactate formation to drive histone K_L‐la_, thus affecting chromatin availability at key developmental loci such as *Pdgfra* and governing craniofacial morphogenesis [126]. During embryonic brain development, microglial metabolic homeostasis regulates lactate accumulation and histone K_L‐la_ at the Lrrc15 promoter. The transcription factor Bach1 restricts glycolytic flux by downregulating HK2 and GAPDH, thus modulating L‐lactate availability and levels of K_L‐la_. Microglia‐derived LRRC15 then engages CD248 to activate Janus kinase (JAK)‐STAT pathway and coordinate astrogenesis [127].

#### 1.7.3 Lactylation at the Nexus of Genome Maintenance

**FIGURE 6:**
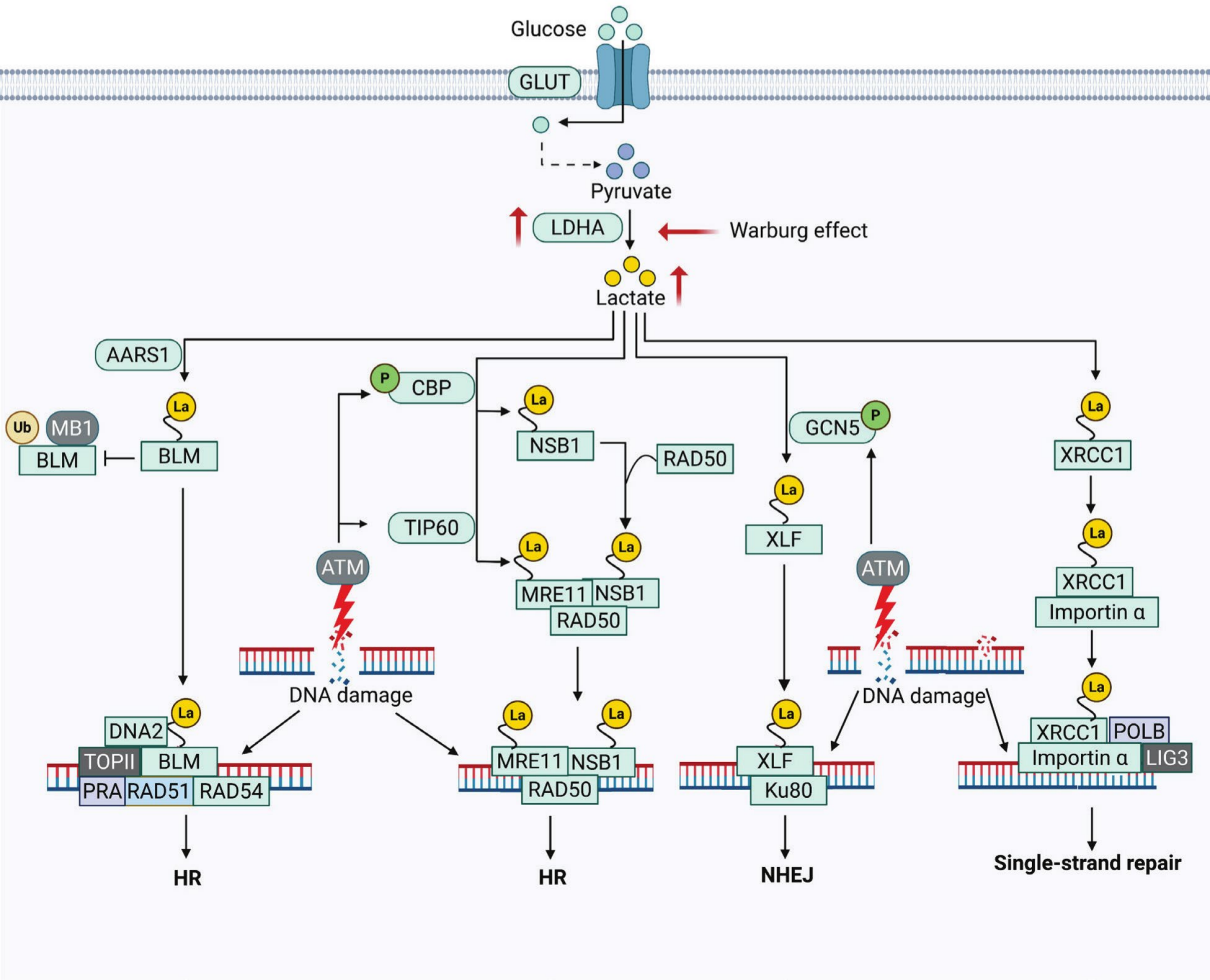
Genome stability. Glycolysis‐fuelled lactylation orchestrates double‐strand break repair via: (i) AARS‐1 mediated BLM lactylation which improves its stability by inhibiting MIB1‐mediated ubiquitination and increasing its interaction with DNA repair factors. (ii) CBP‐mediated MRE11‐K673 and TIP60‐dependent NBS1‐K388 lactylation, stabilizing MRN complex assembly and RAD50 recruitment; (iii) DNA damage triggers ATM‐dependent GCN5 phosphorylation to enhance GCN5‐XLF interaction and XLF lactylation, promoting XLF‐Ku80 binding, XLF recruitment to DSBs and NHEJ activity. (iv) XRCC1 lactylation promoting nuclear import via importin α interaction.

DNA damage, particularly double‐strand breaks (DSBs), occurs continuously in cells due to intrinsic metabolic stress or extrinsic damaging events [128]. There are four pathways responsible for repairing DSBs: homologous recombination (HR), non‐homologous end‐joining (NHEJ), alternative end‐joining, microhomology‐mediated end‐joining (MMEJ) and single‐strand annealing (SSA) [129]. The normal functions of these pathways are crucial for genome stability and precise gene transmission [129]. Non‐histone K_L‐la_ has emerged as an important regulator of these pathways, particularly HR repair and NEHJ (Figure 6). K_L‐la_ of BLM, a key player in HR repair, enhances its interactions with RAD54 and other HR‐associated proteins, thereby facilitating HR repair [3]. Likewise, K_L‐la_ modulates the components of the MRE11–RAD50–NBS1 (MRN) complex, a critical mediator of HR. TIP60‐mediated NBS1‐K388la stabilizes MRN complex assembly and RAD50 recruitment [74], whereas CBP‐catalysed L‐lactylation of MRE11 enhances DNA binding and resection capacity [67]. Consistently, lactylated XLF‐K288 regulates NHEJ [43]. Mechanistically, DNA damage triggers ATM‐dependent GCN5 phosphorylation to enhance GCN5‐XLF interaction and XLF‐K288la, promoting XLF‐Ku80 binding, XLF recruitment to DSBs and NHEJ activity [43].

In other repair pathways, glycolysis‐driven lactate production promotes K_L‐la_ of XRCC1, promoting its bridging with importin α and accelerating its nuclear transport for efficient DNA repair [130]. However, in tumour cells, this metabolic‐epigenetic crosstalk mediated genomic stability, contributes to therapy resistance [3, 43, 67, 74] (Figure 9).

#### 1.7.4 Metabolic Regulation and Energy Homeostasis

**FIGURE 7:**
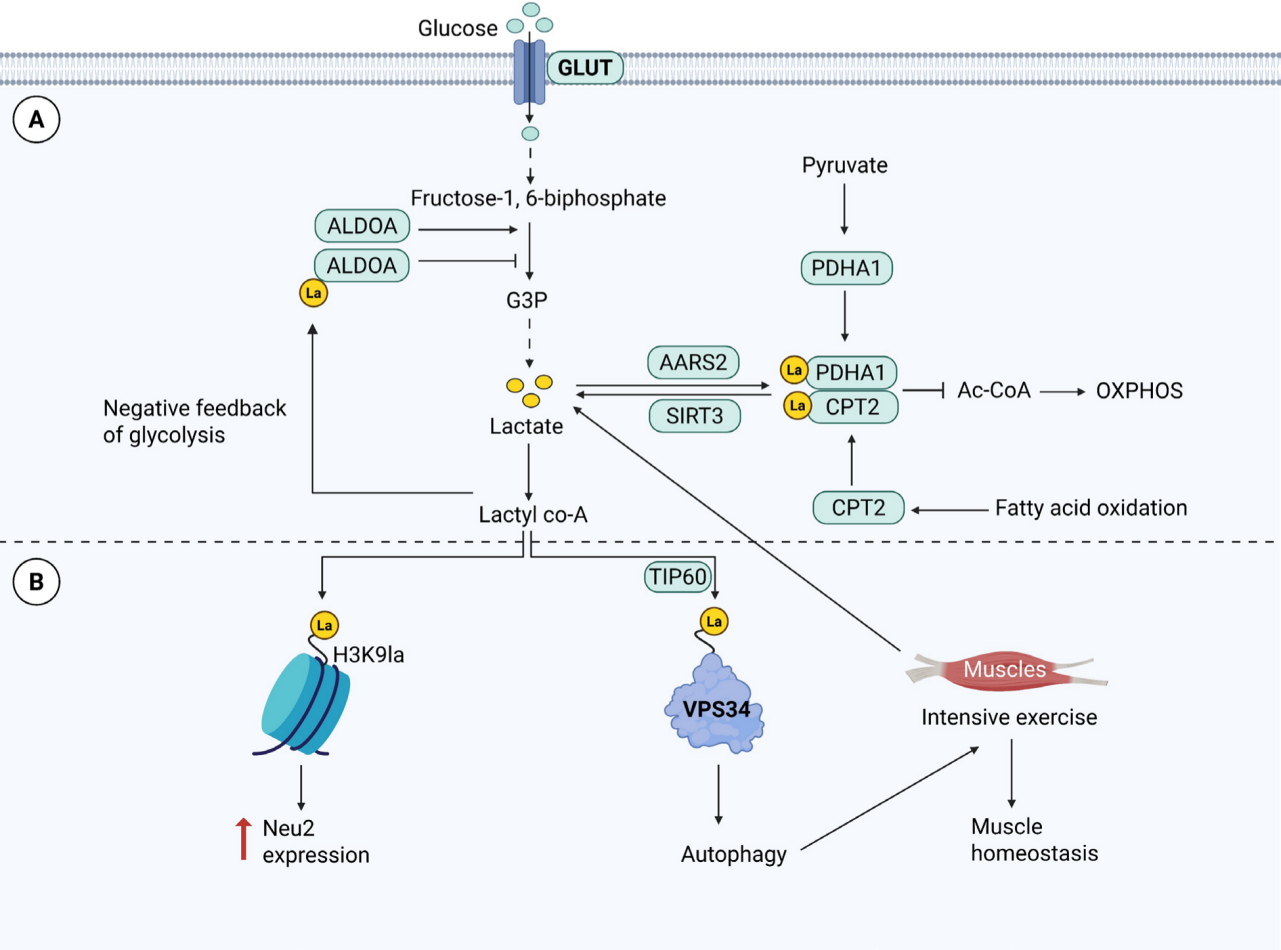
Lactylation coordinates metabolic adaptation and muscle homeostasis. (A) Metabolic regulation: Hyperactive glycolysis triggers ALDOA lactylation (at K147), establishing a feedback brake on glycolytic flux. Hypoxia‐induced lactate promotes AARS2‐mediated lactylation of mitochondrial proteins (PDHA1‐K336, CPT2‐K457/458) to suppress OXPHOS by limiting acetyl‐CoA flux from pyruvate and FA oxidation, while SIRT3‐dependent delactylation reactivates oxidative metabolism upon reoxygenation. (B) Muscle adaptation: Exercise‐derived lactate induces Vps34 lactylation to activate autophagy, maintaining proteostasis during physical stress. Concurrently, H3K9la‐mediated Neu2 upregulation drives myogenic differentiation, with lactate transport blockade impairing muscle regeneration.

Non‐histone K_L‐la_ serves as a central metabolic rheostat, dynamically coordinating cellular energy homeostasis through direct modification of core metabolic enzymes across glycolysis, the pentose phosphate pathway, fatty acid (FA) oxidation and amino acid metabolism [15, 37, 131]. Strikingly, glycolytic enzymes show particularly prominent lactylation signatures [132], with ALDOA‐K147la functioning as a critical feedback inhibitor—a molecular ‘brake’ that attenuates glycolytic flux under conditions of lactate accumulation [37]. Beyond glycolysis, this metabolic control extends to lipid metabolism, as K_L‐la_ of fatty acid synthase (FASN) suppresses its enzymatic activity, reducing de novo lipogenesis and contributing to exercise‐induced fat loss [133]. In mitochondrial adaptation during hypoxia, K_L‐la_ of PDHA1 and CPT2 actively suppresses OXPHOS by limiting acetyl‐CoA flux from pyruvate and FA oxidation, while delactylation restores oxidative metabolism upon reoxygenation [82] (Figure 7A).

#### 1.7.5 Muscle Homeostasis

K_L‐la_ coordinates exercise‐induced muscle adaptation through dual regulation of autophagy and myogenic programming. Following high‐intensity interval training (HIIT), skeletal muscle and liver exhibit peak K_L‐la_ at 24 h post‐exercise [134], with L‐lactate serving as both a metabolic byproduct and signalling molecule. Mechanistically, L‐lactate produced during HIIT mediates K_L‐la_ of Vps34, which activates autophagic flux [135], thus supporting selective removal of damaged organelles and maintaining homeostasis during physical stress. Alongside, L‐lactate induces myogenic differentiation through H3K9la‐induced transcription of Neu2 [136], while pharmacological blockage of L‐lactate transport or synthesis potently inhibits maturation of myocyte [137] (Figure 7B). Notably, macrophage‐dependent muscle repair is orchestrated by dynamic histone lactylation patterns that temporally control pro‐regenerative transcriptional programs [138]. These findings establish K_L‐la_ as a metabolic sensor translating exercise stress into adaptive responses; a direct regulator of myogenic differentiation and a modulator of immune cell function during tissue repair. Future research should focus on temporal and cell‐type‐specific roles of lactylation during muscle regeneration and myogenesis.

#### 1.7.6 Skeletal Development and Bone Formation

The discovery of K_L‐la_ in human skeletal muscle [139] marked the beginning of understanding its indispensable functions in the skeletal system. During osteoblast differentiation, H3K18la enrichment at the JunB promoter enhances its transcription and commitment of mesenchymal precursors to the osteogenic lineage [140, 141]. This regulation is affected in osteoporosis, where bone marrow stromal cells (BMSCs) exhibit lower histone K_L‐la_ and downregulated expression of osteogenic genes [107, 110, 142]. It is notable that endothelial‐generated L‐lactate can rescue this deficit through K_L‐la_ of BMSCs, restoring their osteogenic efficiency [142]. The K_L‐la_‐osteogenesis axis also extends to craniofacial development, where BMP signalling mediated glycolytic reprogramming and subsequent histone K_L‐la_ in NCCs orchestrates appropriate skeletal patterning [143].

### 1.8 K_L‐la_ in Human Diseases

Growing number of evidence extensively implicates K_L‐la_ in a wide range of human disease. In the following, we discuss the roles and mechanisms of K_L‐la_ in regulation of multiple human pathophysiological conditions.

#### 1.8.1 Cancer

Aberrant K_L‐la_ has been reported in multiple types of cancer, where it modulates diverse biological processes through both histone and non‐histone substrates (Table 1). K_L‐la_ participates in carcinogenesis by regulating tumour‐cell intrinsic oncogenic programs, metabolic and epigenetic reprogramming, immune evasion within the tumour microenvironment (TME) and therapeutic resistance.

**TABLE 1:** Roles of lysine L‐lactylation in human cancers.

Cancer type	Lactylation substrates	Roles	Ref
Breast cancer	H3K18	Stabilizes a metastatic protein network (ZWINT, ECT2, ANLN, EZR) to accelerate proliferation and invasion	[144]
	H3K18	Promotes MYC expression, suppress the c‐MYC–SRSF10 pathway and enhances proliferation and spheroid formation	[145]
	H4K12	Inversely correlates with survival	[146]
	p53	Prevents liquid like phase separation and DNA binding, enhances tumour progression	[18]
	RCC2‐K124	Upregulates of MAD2L1, drives tumorigenicity	[49]
Bladder cancer	H3K18	Regulates PRKN‐mediated mitophagy to enhance M2 macrophage polarization	[147]
	H3K18	Induces cancer progression and metastasis via SLC6A14/Glutamine metabolism	[61]
	H3K18	Upregulates transcription factor (TF) Y‐box binding protein 1 (YBX1) and YY1 expression and induces cisplatin resistance	[148]
	YTHDC1	Promotes RNF183‐mediated YTHDC1 ubiquitination, decrease NECTIN4 expression and EV responsiveness	[54]
	BML	Facilitates DNA repair and promotes therapy resistance	[3]
Colorectal cancer	Histone	Facilitates stemness of colon cancer cells	[50]
	HEK9	Facilitates the expression of cholesterol transporter GRAMD1A, promotes cancer progression	[51]
	H3K18	Facilitates the expression of autophagy enhancer protein (RUBCNL), promotes therapeutic resistance	[9]
	H3K18	Promotes carcinogenesis	
	H3K18	Activates transcription of MMP9, promotes liver metastasis in CRC	[59]
	H3K18	Enhances the levels of CXCL1 and CXCL5 expression and promotes cancer metastasis	[149]
	H4K12	Enhances the expression of GCLC, inhibits ferroptosis and promotes chemoresistance of colorectal cancer stem cells	[150]
	eEF1A2‐K408	Increases translation efficiency, promotes biosynthesis and carcinogenesis	[42]
	NOL6	Promotes tumour progression via STAMBP‐induced deubiquitination of YY1 and transcriptional upregulation of c‐Myc	[151]
	GCLM, HDAC1	Confers ferroptosis resistance	[52, 76]
	ANTXR1	Facilitates cancer stemness and promotes resistance for oxaliplatin	[115]
	β‐Catenin	Promotes the expression of β‐catenin expression, enhances proliferation and stemness	[114]
	CEACAM6	Stabilizes CEACAM6, promotes proliferation and chemoresistance	[152]
	METTL3 K281/K345	Promotes immunosuppression of tumour‐infiltrating myeloid cells	[153]
Cervical cancer	H3K18	Activities the expression of ARG1, promotes M2 polarization, drives cancer progression	[154]
	H3K18	Drives GPD2, reinforces M2 polarization, promotes malignant transformation of Cervical Cancer progression	[155]
	H3K14	Induces transcriptional activation of oncogenes, initiation and progression of cancer	[102]
	DCBLD1‐K172	Activates pentose phosphate pathway, promotes proliferation, migration and metastasis	[156]
	G6PD K45	Suppresses in vivo/in vitro cell proliferation	[131]
Glioblastoma	H3K9	Activates transcription of LUC7‐like 2 and promotes TMZ resistance	[157]
	H3K18	Upregulates CD39/CD73/CCR8, promotes adenosine + Treg‐mediated immunosuppression and CAR‐T resistance	[158]
	H3K18	Enhances tumour necrosis factor superfamily member 9 (TNFSF9) expression, induces M2 macrophage polarization	[21]
	H3K18	Increases USP4 expression, stabilizes and activates and activation ANXA2, promotes GSCs maintenance and radio resistance	[56]
	H3K14/18	Activation of an immuno‐evasion transcriptomic profiling	[66]
	XRCC1 K247	↑Binding affinity of nuclear transport protein importin α ↑Chemo‐radiotherapeutic resistance	[130]
Gastric cancer	METTL16	Enhances cuproptosis via m6A‐modification on FDX1 mRNA	[159]
	NBS1 K388	Facilitates DNA repair and exerts resistance to chemotherapy	[74]
	NSUN2	Increases GCLC‐dependent glutathione synthesis and confers cancer cell resistance for ferroptosis	[58]
	YAP K90/TEAD1 K108	Promotes YAP‐TEAD1 expression, nuclear localization and cell proliferation	[78]
Head/neck squamous cell carcinoma Hepatocellular carcinoma	H3K9	Promotes CD8+ T cell dysfunction and poor response to immunotherapy	[160]
	H3K9, H3K56	Aggressive tumour traits	[161]
Lung cancer	H2BK58	Induces NDRG1‐associated transcriptional reprogramming, mediates senescence resistance, promotes tumour progression	[162]
	Histone	Modulates lipid metabolism remodelling and enhances tumorigenesis	[65]
	ABCF1‐K430l	Transcriptionally activates HIF1 signalling and increases cancer progression	[163]
	ALDOA K230/K322	Modulates DDX17 activity and enhances CSC self‐renewal	[164]
	MOESIN	Promotes TGF‐β/SMAD3 signalling to enforce immunosuppression	[165]
	IGF2BP3	Metabolism reprogramming and RNA m6A—modification, resistance against lenvatinib	[166]

#### 1.8.2 K_L‐la_ in Tumour‐Cell Intrinsic Oncogenic Programs

Evidence highlights that K_L‐la_ promotes tumour progression by regulating proliferation, invasion, metastasis, stemness and cellular plasticity. Histone K_L‐la_ induces oncogenic transcriptional programs across different malignancies. In breast cancer, H3K18la enrichment at the c‐Myc promoter activates serine/arginine‐rich splicing factor 10 (SRSF10)‐dependent splicing program that generates pro‐survival MDM4 and Bcl‐x isoforms [145]. H2BC9‐K44la promotes Wnt7b transcription and activates Wnt/β‐catenin signalling in oesophageal squamous cell carcinoma (ESCC) [167]. Similarly, in glioblastoma (GBM), epidermal growth factor receptor (EGFR)‐driven formation of the ACSS2‐KAT2A complex promotes lactyl‐CoA‐dependent histone K_L‐la_, leading to activation of Wnt/β‐catenin, NF‐κB, and P_d‐L1_ transcriptional programs that support tumorigenesis [66]. Moreover, H3K18la‐induced RUNX2 expression activates PI3K‐AKT signalling in laryngeal squamous cell carcinoma (LSCC) [168]. In CRC, KRAS‐driven lactate production increases H3K9la at the GRAMD1A promoter, enhancing chromatin accessibility and promoting tumour growth and metastasis [51]. Similarly, H3K9la and H3K56la sustain proliferation and self‐renewal of liver cancer stem cells in HCC [161].

Beyond histones, non‐histone K_L‐la_ directly regulates oncogenic signalling pathways. L‐lactylation of β‐catenin enhances its stability and potentiates Wnt‐driven oncogenesis in CRC [114], whereas AARS1‐mediated L‐lactylation of YAP–TEAD1 promotes transcriptional activation and proliferation in gastric cancer [159]. Lactylated ALDOA sustains liver cancer stem cell self‐renewal through modulation of DDX17 activity [164]. Moreover, lactylation‐dependent stabilization of NOL6 facilitates STAMBP‐mediated deubiquitination of YY1 and consequent activation of c‐Myc transcription, thereby promoting CRC progression [151]. Collectively, these findings establish K_L‐la_ as a critical regulator of oncogenic signalling, tumour stemness and cellular plasticity.

#### 1.8.3 K_L‐la_ ‐Mediated Metabolic and Epigenetic Reprogramming

Besides directly regulating oncogenic pathways, K_L‐la_ contributes to tumour progression through metabolic and epigenetic reprogramming. H3K18la has been shown to implicate in oncogenic transcriptional reprogramming, promoting hypoxia‐driven metabolic adaptation via solute carrier family 6 member 14 (SLC6A14) upregulation [61]. Histone lactylation‐induced expression of YTHDC1 associates with HCC progression via lipid metabolism remodelling [65]. Emerging evidence also highlights a role for K_L‐la_ in epitranscriptomic regulation. Copper‐induced L‐lactylation of METTL16 enhances its m6A methyltransferase activity in gastric cancer [159], while K_L‐la_‐driven IGF2BP3‐PCK2‐SAM‐m6A signalling promotes lenvatinib resistance in HCC [166]. Likewise, K_L‐la_ of RBM15 enhances N6‐methyladenosine (m6A)‐dependent oncogenic signalling in lung cancer [169]. Together, these studies suggest that K_L‐la_ integrates metabolic, transcriptional and post‐transcriptional regulatory networks to facilitate tumour adaptation.

#### 1.8.4 K_L‐la_ in the Tumour Microenvironment and Immune Evasion

Lactate has been shown to promote regulatory T cell (Treg) function in the TME [170, 171], while inhibiting monocyte differentiation into dendritic cells [172]. Lactate also induces macrophage polarization towards an immunosuppressive M2‐like phenotype [173]. Thus, lactate exerts vital functions in establishing the immunosuppressive characteristics of the TME. Emerging studies revealed that K_L‐la_ is a critical mechanism linking tumour‐derived lactate to immune suppression within the TME. In the following, we discuss the mechanisms by which lactate induced‐K_L‐la_ regulates tumour growth and immune evasions.

K_L‐la_ influences the behaviour of immune cells in TME. In CRC, KRAS mutations promotes the glycolysis, leading to enhanced lactate production. This lactate drives histone K_L‐la_ in tumour‐specific cytotoxic T cells (CTLs), upregulating circular RNA *circATXN7* expression [174]. CircATXN7 sequesters NF‐κB in the cytoplasm, promoting CTLs susceptibility to activation‐induced cell death [174].

Regulatory T (Treg) cells play a pivotal role in sustaining the immunosuppressive tumour microenvironment [175]. Lactate promotes Treg cell stability and function through L‐lactylation of membrane–organizing extension spike protein (MOESIN) [165]. Mechanistically, L‐lactylation enhances the interaction of MOESIN with TGF‐β receptor I, leading to activation of SMAD3 signalling and increased FOXP3 expression, thereby further enhancing Treg‐mediated immunosuppression [165]. Supporting these observations, elevated levels of lactylated MOESIN in Treg cells were associated with decreased responsiveness to anti‐PD1 therapy in HCC patients [165].

Additionally, K_L‐la_ regulates the function of immunosuppressive myeloid cells. Within the GBM TME, glycolytic reprogramming in monocyte‐derived macrophages leads to intracellular lactate accumulation and histone lactylation which induces the expression of IL‐10, a key factor in T cell suppression [176]. Similarly, in CRC, tumour‐derived lactate promotes H3K18la‐dependent expression of METTL3 in tumour‐infiltrating myeloid cells. METTL3 then enhances m6A modification of JAK1 mRNA, promoting JAK1–STAT3 signalling and reinforcing the immunosuppressive capacity of these cells [153].

K_L‐la_ also promotes polarization of macrophages towards a protumorigenic M2 phenotype. In cervical cancer, tumour‐derived lactate enhances the expression of ARG1 and GPD2 through H3K18la in tumour‐associated macrophages, thereby driving M2 polarization and immune evasion [154, 155]. Recent studies further revealed that H3K18la also promotes M2 macrophage polarization and tumour progression in bladder cancer and head and neck squamous cell carcinoma (HNSCC) [147, 177].

#### 1.8.5 K_L‐la_ and Therapeutic Resistance

Therapeutic resistance represents one of the most rapidly expanding areas of lactylation research. K_L‐la_ promotes resistance to chemotherapy, radiotherapy and targeted therapies. Several studies demonstrated roles for K_L‐la_ in resistance to cisplatin and enfortumab vedotin (in bladder cancer) [54, 148], oxaliplatin (in CRC) [115], sorafenib and lenvatinib (in HCC) [166, 178, 179, 180] and immunotherapies (in different cancers) [160, 165, 174]. K_L‐la_ promotes therapeutic resistance through several different mechanisms. First, K_L‐la_ (non‐histone; BLM, XRCC1, XLF) enhances DNA damage repair to confer therapeutic resistance in diverse cancer types [3, 43, 130]. Second, K_L‐la_ (histone; H4K12 and non‐histone; HDAC1, GCLM) drives therapeutic resistance in CRC through suppression of ferroptosis [52, 76, 150]. Third, lactylation‐dependent activation of rubicon‐like autophagy enhancer (RUBCNL) promotes autophagy‐mediated therapeutic resistance [14]. Collectively, these findings positioned K_L‐la_ as a multifaceted driver of therapeutic resistance through modulation of DNA repair, ferroptosis, autophagy and immune evasion.

#### 1.8.6 Viral Infections

**FIGURE 8:**
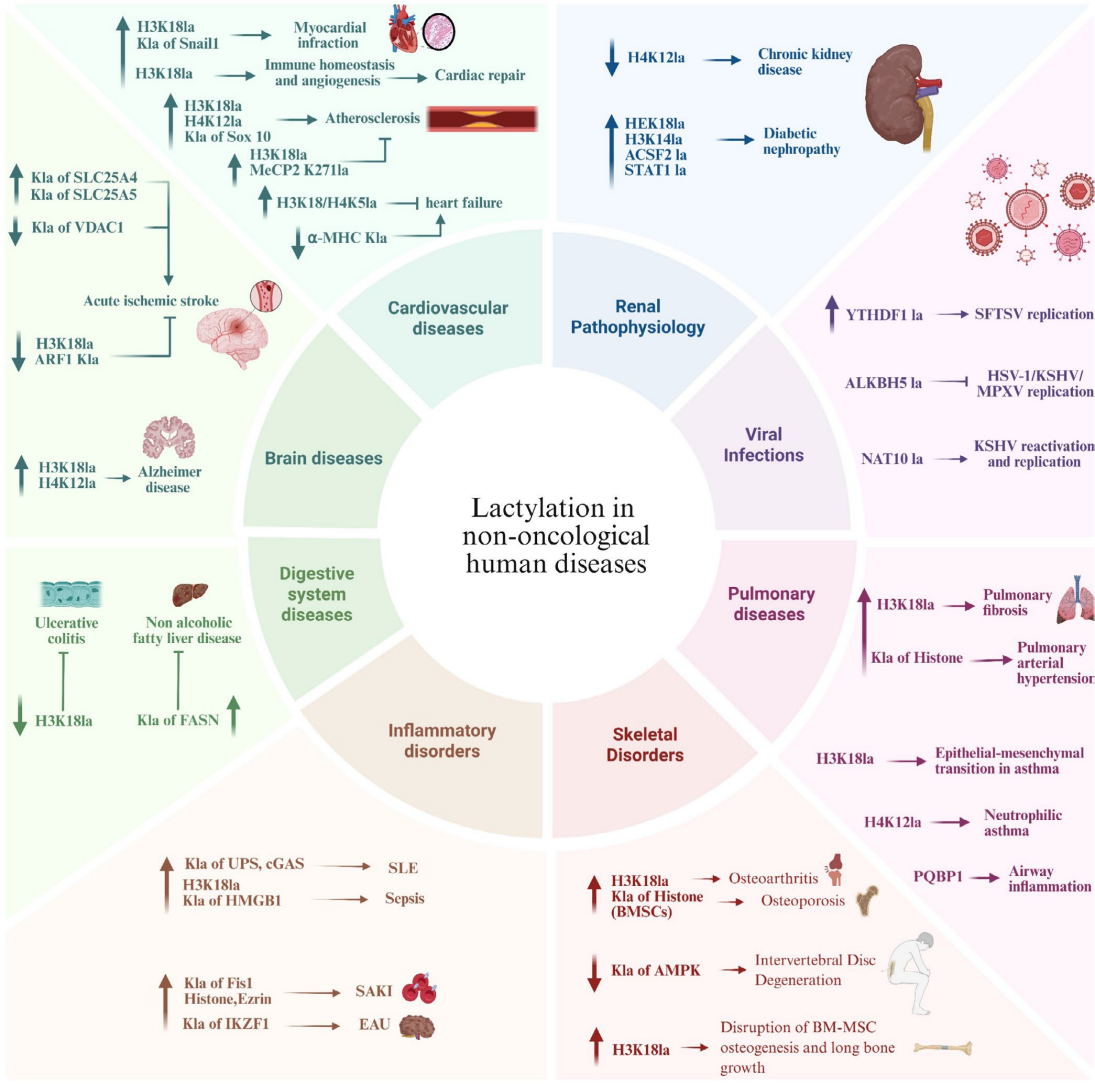
The multifaceted roles of lysine lactylation in human diseases. Lysine L‐lactylation exerts broad yet context‐dependent effects, including dual roles in cardiovascular diseases, regulatory functions in brain, digestive and inflammatory disorders, contributions to skeletal and pulmonary pathologies, and involvement in viral infections and renal diseases.

Non‐histone K_L‐la_ has appeared to play a context‐dependent role in viral infections (Figure 8). Severe fever with thrombocytopenia syndrome virus (SFTSV)‐induced K_L‐la_ of m6A reader YTHDF1 facilitates viral replication [181], while L‐lactylation of the m6A demethylase AlkB homologue 5 (ALKBH5) increases innate antiviral immunity by promoting interferon‐beta (IFN‐β) mRNA biogenesis and restricting viral replication [77]. ATAT1‐mediated K_L‐la_ of NAT10 enhances N4‐acetylcytidine modification on tRNASer‐CGA‐1‐1 to promote oncogenic DNA virus Kaposi's sarcoma‐associated herpesvirus (KSHV) reactivation [75]. While these findings exhibit that K_L‐la_ of host proteins can either enhance antiviral immunity or facilitate viral replication depending on the substrate involved, a recent study revealed that K_L‐la_ can also directly modify viral proteins to influence viral infectivity. For instance, K_L‐la_ of the SARS‐CoV‐2 spike (S) protein promotes its interaction with the host receptor angiotensin‐converting enzyme 2 (ACE2) or the host protease transmembrane protease serine 2 (TMPRSS2), thus facilitating viral entry and infection [44]. This study delineates new insights for the development of antiviral therapeutics. However, specific enzymes regulating S protein K_L‐la_ remain undefined and warrant further investigations.

#### 1.8.7 Neurodegenerative Diseases

##### 1.8.7.1 Alzheimer's Disease (AD)

AD is a chronic neurodegenerative disorder characterized by extracellular amyloid‐β (Aβ) deposition, intracellular tau pathology and tenacious neuroinflammatory responses [182]. Both histone and non‐histone K_L‐la_ contribute to disease pathogenesis (Figure 8). Elevated levels of histone K_L‐la_ have been observed in brain tissues from AD patients and 5XFAD mice, with H4K12la promoting the transcription of glycolysis‐associated genes, reinforcing a self‐sustaining glycolysis–PKM2–H4K12la signalling circuit that contributes to microglial dysfunction [183]. In addition, the microbial small RNA P.G_45033 enhances Aβ accumulation by stimulating glycolytic metabolism and enhancing histone L‐lactylation in macrophages [184]. Metabolic disruption caused by isocitrate dehydrogenase 3β (IDH3β) deficiency further promotes lactate accumulation, leading to increased H4K8la, H4K12la and H3K18la levels. Notably, the transcription factor PAX6 acts downstream of IDH3β and suppresses its expression, establishing a regulatory network involving IDH3β, lactate production, lactylation and PAX6 that promotes AD‐like pathological alterations [185]. H3K18la upregulation in senescent microglia has also been implicated in accelerating brain aging and AD‐related pathology through activation of NF‐κB signalling [186]. Beside these effects, pan‐K_L‐la_ influences hippocampal expression of the *Kmt2a* gene, thereby impairing synaptic and cognitive function [187]. Furthermore, lactylated non‐histone tau protein has been shown to potentiate ferroautophagy‐ and ferroptosis‐associated pathways via MAPK signalling, resulting in microglial activation and neuroinflammatory responses [188].

##### 1.8.7.2 Parkinson's Disease

Parkinson's disease (PD) is characterized by the progressive degeneration of dopaminergic neurons [189]. Emerging evidence indicates that metabolic reprogramming towards increased glycolysis contributes to disease pathology by endorsing microglial activation. Importantly, elevated glycolytic flux promotes H3K9la, which induces pro‐inflammatory responses in microglia, thereby fostering neuroinflammation and accelerating neurodegeneration associated with PD [57]. Notably, pharmacological inhibition of glycolysis using 2‐deoxy‐D‐glucose (2‐DG) reduces lactate accumulation and mitigates disease‐related pathology in experimental mouse models, underscoring the therapeutic potential of targeting glucose metabolism and lactylation‐associated signalling pathways in PD [57].

#### 1.8.8 Cardiovascular Diseases

Cardiovascular diseases (CVDs) are the leading cause of death worldwide [190], with K_L‐la_ emerging as a pivotal epigenetic and metabolic regulator in disease pathophysiology (Figure 8). For example, histone K_L‐la_‐induced GLI3 activation has been reported to drive macrophage M1 polarization and exosomal protein SERPINE1 release, thereby promoting abdominal aortic aneurysm (AAA) progression [191]. In the following, we will elucidate the roles and mechanisms of both lactylated histone and non‐histone proteins across major forms of CVDs.

##### 1.8.8.1 Atherosclerosis

Endothelium‐specific solute carrier family 22 member 6 (SLC22A6)‐mediated lactate deposition enhances H3K9la, activating stearoyl‐CoA desaturase 1 (SCD1) transcription and driving endothelial dysfunction and atherosclerosis [192]. Glycolysis‐driven lactate elevation promotes H3K18la at NF‐κB pathway gene promoters, amplifying endothelial inflammation and early plaque formation [193]. Interestingly, Narciclasine has been reported to inhibit this lactylation‐mediated NF‐κB activation, thereby alleviating the initiation of endothelial inflammation and atherosclerosis [193].

In mouse model, non‐histone K_L‐la_, for example, methyl‐CpG‐binding protein 2 (Mecp2)‐K271la epigenetically represses *Ereg* transcription, thereby suppressing EGFR/MAPK signalling pathway and expression of downstream target genes ultimately attenuating atherosclerosis progression [194].

##### 1.8.8.2 MI

Emerging evidence reveals lactate‐mediated K_L‐la_ as a master regulator of post‐MI cardiac repair, exhibiting temporally distinct roles in inflammatory resolution and fibrotic remodelling. Following MI, monocytes undergo metabolic reprogramming where H3K18la upregulates reparative genes (LRG1, VEGF, IL‐10) accelerating M2 polarization and activating cardiac repair [14]. Paradoxically, some endothelial cells undergo EMT post MI, contributing to cardiac fibrosis and exacerbating cardiac dysfunction [195]. Lactate exposure has been shown to elevate K_L‐la_ of snail family transcriptional repressor 1 (Snail1), facilitating its nuclear translocation in endothelial cells and subsequently activating TGF‐β1/Smad2 signalling to promote EMT and cardiac fibrosis [196].

##### 1.8.8.3 Heart Failure (HF)

Reduced K_L‐la_ of α‐myosin heavy chain (α‐MHC) is correlated with disease progression in both mice models and human patients with HF [17]. Mechanistic studies revealed that α‐MHC‐K1897la is critical for its interaction with Titin, that is, crucial for cardiac structure and function maintenance [17]. METTL7B‐induced histone K_L‐la_ in myocardium prevents HF through ameliorating cardiac remodelling [197]. H3K18la promotes pathological cardiac hypertrophy and heart failure by upregulating TGFβ2 and activating PI3K‐AKT‐mTOR signalling [198]. Pharmacological inhibition of PI3K or AKT attenuated this effect, while cardiac‐specific *Tgfb2* knockdown reversed the prohypertrophic effects of histone K_L‐la_ in vivo [198].

##### 1.8.8.4 Pulmonary Hypertension

In hypoxic pulmonary hypertension, mitochondrial reactive oxygen species (ROS)‐driven hypoxia‐inducible factor 1 alpha (HIF‐1α) activation promotes glycolytic reprogramming and lactate accumulation, leading to H3K18 lactylation–dependent transcription of pro‐proliferative genes that drive pulmonary vascular remodelling [199]. These findings position histone K_L‐la_ as a therapeutic target in pulmonary hypertension.

#### 1.8.9 Inflammatory Disorders

Despite recent successes with biopharmaceuticals, inflammatory diseases are the major burden on mankind. A growing number of evidence implicates K_L‐la_ in the pathogenesis of multiple inflammatory diseases. For example, in retinal vascular diseases, microglial lactylated YY1 transcriptionally activates fibroblast growth factor 2 (FGF2) to promote pathological angiogenesis [200]. Here in this section, we discuss K_L‐la_ in primary inflammation conditions.

##### 1.8.9.1 Neuroinflammation

Neuroinflammation is dynamically regulated by K_L‐la_, which differentially modulates microglial and astrocytic phenotypes in a context‐dependent manner. Microglia exhibit a biphasic K_L‐la_ response: acute inflammatory triggers (Cgas‐la, YY1‐la) promote M1 polarization via NF‐κB activation [201, 202], while chronic conditions (spinal cord injury, exercise) induce H4K12la‐mediated M2 reparative transitions [203]. Astrocytes demonstrate K_L‐la_‐driven neuroprotection, where BRD4‐dependent H4K12la suppresses A1 polarization [204].

##### 1.8.9.2 Sepsis

Sepsis‐associated hyperlactatemia (> 2 mmol/L) transcends its diagnostic role by fuelling pathogenic lactylation modifications that exacerbate multi‐organ dysfunction: in macrophages, p300/CBP‐mediated high mobility group box‐1 (HMGB1) K_L‐la_ and acetylation drive nuclear‐to‐cytoplasmic translocation, amplifying endothelial barrier disruption [38]; renal tubular cells exhibit mitochondrial fission 1 protein (FIS1)‐K20la‐induced mitochondrial fission, depleting ATP and promoting apoptosis [205]; while alveolar epithelia undergo METTL3‐la‐dependent ACSL4 m6A stabilization, triggering ferroptotic lung injury [206]. This organ‐specific L‐lactylation cascade, spanning epigenetic (HMGB1‐la), organellar (FIS1‐la) and epitranscriptomic (METTL3‐la) regulation, positions lactate as both a biomarker and mechanistic driver of septic shock, with MCT1 inhibition emerging as a potential therapeutic strategy to disrupt lactylation‐mediated tissue damage (Figure 8).

##### 1.8.9.3 Autoimmune Diseases

Elevated lactate in autoimmune diseases fuels pathogenic L‐lactylation modifications that disrupt immune homeostasis: in systemic lupus erythematosus (SLE), HIF‐2α‐mediated glycolytic shift induces UPS subunit lactylation, impairing mitochondrial clearance and triggering cGASla‐dependent IFN‐I production via membrane‐associated RING‐CH‐type finger 5 (MARCHF5) evasion [207, 208]; in autoimmune uveitis, IKAROS family zinc finger 1 (IKZF1)‐K164la enhances TH17 differentiation by strengthening promoter binding at Runx1/Tlr4/Il2/Il4 loci [209]. These findings reveal lactylation as a unifying mechanism linking metabolic dysregulation (HIF‐2α stabilization, glycolysis) to autoimmune effector functions (IFN‐I, TH17), with therapeutic potential demonstrated by glycolysis inhibition ameliorating both SLE chimeras [208] and experimental autoimmune uveitis (EAU) models [209] (Figure 8).

#### 1.8.10 Pulmonary Diseases

##### 1.8.10.1 Pulmonary Fibrosis

Pulmonary fibrosis (PF) is characterized as a chronic respiratory disorder in which gradual replacement of lung tissue with fibrotic tissues happens. Recent reports have revealed that human alveolar macrophages have got upregulated lactate levels may involve in enhanced K_L‐la_ [39]. Indeed, histone K_L‐la_ mediates a great expression of pro‐fibrotic‐related factors at transcriptional level, and has a key role in PF progression [39]. For instance, a study by Cui and colleagues confirmed lactate production in myofibroblasts enriches H3K18la in the promoter regions of pro‐fibrotic genes, particularly *ARG1*, *PDGFA*, *VEGFA* and *THBS1*. An elevated transcriptional activity of these genes may enhance pro‐fibrotic properties of alveolar macrophages, exacerbating PF [39]. Similarly, another study claimed that extracellular lactate from myofibroblasts has raised overall K_L‐la_ and H3K18la contents through MCT1. H3K18la facilitates the progression of arsenic (As)‐related idiopathic PF via the YTHDF1/m6A/NREP signalling cascade [210] (Figure 8).

##### 1.8.10.2 Asthma

Asthma is a chronic inflammatory airway disease characterized by airway hyperresponsiveness, remodelling and dysregulated immune responses [211]. Metabolic reprogramming and lactate accumulation have emerged as key contributors to asthma pathogenesis through both histone and non‐histone K_L‐la_ (Figure 8). In an experimental asthma, activation of the HIF‐1α–glycolysis axis elevates lactate production and global protein lactylation in lung tissues, particularly in macrophages. Interestingly, dexamethasone can suppress glycolysis, diminish lactate production and K_L‐la_ levels, concomitantly attenuating airway inflammation and pyroptosis [212]. Histone K_L‐la_ specifically regulates immune remodelling in asthma. H3K18la is upregulated in the lungs of asthmatic mouse models and functionally promotes DPP4 transcription and Th17 differentiation, thereby facilitating epithelial–mesenchymal transition and airway remodelling [213]. H4K12la enhances CXCL1 and CXCL2 expression to drive neutrophil recruitment in severe neutrophilic asthma [214]. Besides histones, non‐histone K_L‐la_ also plays crucial roles in disease pathogenesis. For example, lactylated polyglutamine‐binding protein 1 (PQBP1) inhibits the PRMT5/WDR77 complex, relieving transcriptional repression of pro‐inflammatory genes and establishing a metabolic–epigenetic circuit that amplifies airway inflammation [215]. In addition, ATP6V1B2 is recognized as a key lactylation substrate in airway epithelial cells, where lactylation at K108/K109 disrupts V‐ATPase assembly and function, leading to lysosomal dysfunction, membrane permeabilization, mitochondrial ROS production and Caspase‐8/3–GSDME‐mediated pyroptosis [216]. Collectively, these findings position K_L‐la_ as a central regulator of immune dysregulation, epithelial injury, pyroptosis and airway remodelling in asthma, highlighting lactylation‐associated pathways as promising therapeutic targets for this disease.

#### 1.8.11 Renal Pathophysiology

##### 1.8.11.1 Diabetic Nephropathy

Diabetic nephropathy (DN) is a frequent complicated condition in diabetes and a leading cause of death among diabetic patients [217]. Ferroptosis is considered a critical attribute of DN, inducing the death of renal cells [218]. ELOVL FA elongase‐5 (ELOVL5) is a member of the ELOVL family and is involved in ferroptosis [219]. It has been documented that L‐lactylation of H3K18 and STAT1 modulates ELOVL5 at the transcriptional level and promotes ferroptosis in a DN model [79]. ACSF2 and K_L‐la_ facilitate renal tubule injury in diabetes [220]. Furthermore, aging induces a metabolic shift in renal tubular epithelial cells, leading to enhanced lactate production. In diabetic kidney disease (DKD), this increases H3K14la, which promotes *KLF5* expression and subsequently suppresses CDH1, thereby promoting EMT and renal fibrosis [221]. Accordingly, repressing lactate accumulation or histone K_L‐la_ may alleviate tubular fibrotic progression in DKD (Figure 8).

##### 1.8.11.2 Chronic Kidney Disease

Chronic kidney disease (CKD) is a prevailing health issue [222]. Current knowledge about K_L‐la_'s role in CKD is insufficient. A study reported that glycolytic enzyme 6‐phosphofructo‐2‐kinase 3 (PFKFB3) induces aberrant histone K_L‐la_ in renal tubular epithelial cells. Its genetic deletion downregulates H4K12la and NF‐κB signalling, and mitigates renal inflammation and fibrosis, suggesting a novel therapeutic target in CKD [223].

#### 1.8.12 Skeletal Disorders

K_L‐la_ is precisely associated with metabolic homeostasis in skeletal tissues and functions in a context‐dependent manner, demonstrating its potential as a therapeutic target in musculoskeletal disorders (Figure 8). Chronic intermittent hypoxia promotes H3K18la, impairing osteogenic differentiation of BMSC and disrupting long bone growth [224]. In osteoarthritis, LDHA‐driven H3K18la promotes disease progression by activating glycolysis‐associated transcription, including *TPI1* expression [225]. In osteoporosis, downregulated systemic lactate levels are observed, whereas exercise‐mediated lactate elevation promotes histone K_L‐la_ in MSCs and strengthens osteogenesis, substantiating a protective role [142]. In intervertebral disc degeneration, altered amino acid and lactate metabolism is accompanied by increased K_L‐la_, while glutamine supplementation suppresses glycolysis, reduces AMPKα lactylation and prevents degenerative changes [226]. In tendons, K_L‐la_ is associated with dysregulated cholesterol metabolism and extracellular matrix remodelling, reflecting tissue degeneration [227].

#### 1.8.13 Digestive System Diseases

K_L‐la_ has appeared as an important regulator of digestive diseases, including non‐alcoholic fatty liver disease (NAFLD) and ulcerative colitis (UC) (Figure 8). In UC, histone K_L‐la_ predominantly plays pathogenic roles. Inhibition of H3K18la suppresses macrophage pyroptosis, normalizes intestinal immune homeostasis and promotes the function of the mucosal barrier [228]. Consistently, Ge Gen Qin Lian Tang (traditional Chinese medicine) alleviates colitis by downregulating lactate production and histone K_L‐la_, thereby suppressing M1 macrophage polarization and inflammatory responses [229]. In NAFLD, mitochondrial pyruvate carrier 1 (MPC1) regulates hepatic lactate availability and promotes fatty acid synthase (FASN) K673 lactylation, which inhibits FASN activity and attenuates hepatic lipid accumulation, suggesting protective effects [230].

#### 1.8.14 Other Diseases

Histone K_L‐la_ is closely associated with pregnancy‐related diseases. Placental hypoxia in preeclampsia enhances lactate production in trophoblasts, mediating histone K_L‐la_ and upregulating fibrosis‐related genes such as *FN1* and *SERPINE1* [231]. Higher histone K_L‐la_ links metabolic dysregulation to transcriptional reprogramming in gestational diabetes mellitus, promoting the expression of genes involved in cell proliferation and diabetes‐associated signalling pathways [232]. Histone K_L‐la_ is also implicated in skin injury and disease, with elevated levels in hypertrophic scar tissues upregulating *SLUG* and downregulating *PTEN*, thereby suppressing autophagy and promoting collagen deposition and fibroblast viability [233]. Zhao et al. found lower ADIPOQ and H3K18la levels in psoriatic skin. H3K18la supports ADIPOQ promoter binding, and its reduction downregulates *ADIPOQ* expression, alleviating psoriasis progression [234].

### 1.9 Therapeutic Implications of K_L‐la_

Modulation of both histone and non‐histone K_L‐la_ has shown promised therapeutic potential for multiple diseases. Therapies can be classified into four types: (1) targeting metabolic enzymes (3), (2) inhibition of lactate transport and sensing, (3) targeting enzymes that regulate K_L‐la_ and (4) targeting lactylation sites directly.

#### 1.9.1 Targeting Metabolic Enzymes

Targeting metabolic enzymes to modulate K_L‐la_ offers potential therapeutics. For example, treatment with PKM2 inhibitors (shikonin or compound 3 K) reduces H4K12la and Aβ burden and attenuates microglial activation in AD mice [183]. In bladder cancer, inhibition of PKM2 (by using Mannose) and subsequently K_L‐la_ induces pyroptosis and activates antitumor immune responses [71]. In NSCLC, natural product fargesin targets PKM2 to interfere with H3 K_L‐la_ and inhibit carcinogenesis [235]. Beyond PKM2, LDHA inhibitors also demonstrate significant therapeutic efficiency. For instance, sodium oxamate‐mediated suppression of H3K18la enhances CAR‐T efficacy against GBM [158] while concurrently restraining LSSC progression by suppressing proliferation and migration and promoting apoptosis [168]. Stiripentol disrupts NBS11 lactylation and subsequently DNA repair to overcome chemoresistance [74], and (R)‐GNE‐140 blocks H3K9la‐driven myogenesis [136]. Moreover, combining LDHA inhibitor GSK2837808A and anti‐PD‐1 enhances antitumour efficiency by delactylating MOESIN in Tregs [165].

These strategies highlight K_L‐la_ as a druggable node, with enzyme inhibitors offering precision control over pathogenic modifications. However, cell‐specific delivery is required to balance on‐target efficacy and metabolic toxicity. Additionally, the bidirectional relationship between lactate metabolism and the TME suggests that merely ‘inhibiting lactate production’ may lead to side effects. Instead, precise regulation of the spatial expression and temporal activity of lactate represents a more promising strategy.

#### 1.9.2 Inhibition of Lactate Transport and Sensing

Targeting lactate shuttling via transporter has also opened therapeutic avenues. For example, inhibition of lactate influx via MCT1/4 inhibitor 7ACC1 decreases ANTXR1‐K453la and increases antitumor efficiency of oxaliplatin in CRC [115]. MCT1 inhibitor AZD3965 prevents lactate export and elevates intracellular lactate levels [236]. C2C12 cells treated with AZD3965 exhibited accumulated lactate and histone K_L‐la_, with promoted muscle differentiation in mouse myoblast [136]. MCT4 inhibitor VB124 has been shown to enhance intracellular lactate and K_L‐la_ of α‐MHC in cardiomyocytes, preventing HF [17]. Receptor modulation, for example, GPR81/132 inhibition, remains limited by poor lactate specificity [237, 238, 239].

#### 1.9.3 Targeting Enzymes Regulating K_L‐la_

Inhibitors of lactyltransferases (p300/AARS1/AARS2) have been reported to decrease K_L‐la_ levels, representing another potential strategy. For example, *p300* inhibitors (C646, A‐485) suppress pathogenic K_L‐la_ events, with A‐485 potently blocking lactylated YY1‐driven retinal microglial inflammation [200], while C646 inhibits *H3K9la*‐mediated myogenesis [136]. β‐Alanine disrupts *AARS1*‐mediated K_L‐la_ and *p53* suppression by competing with L‐lactate for AARS1 binding [18]. Moreover, β‐alanine treatment reduces H3K18la and ELOVL5 expression, suppresses ferroptosis, decreases renal injury and improves renal dysfunction in DN mice and hyperglycaemic cells [79]. Another study reported that β‐alanine occupies the alanine‐binding pocket of AARS2, attenuating K_L‐la_ of PDHA1 and CPT2 and boosting exercise capacity in a mouse model [82]. L‐Alanine also competitively inhibits AARS1–L‐lactate interaction, thereby suppressing K_L‐la_ [78, 80].

Targeting delactylases (SIRT1‐3/HDAC1‐3) also opened a therapeutic avenue. K_L‐la_ of METTL16 enhances cuproptosis in GC and inhibition of SIRT2 by AGK2 further supports METTL16 lactylation to promote cuproptotic cell death [159]. SIRT3 activation by Honokiol inhibits K_L‐la_ of CCNE2 in HCC [96]. Moreover, HDAC inhibitors (sodium butyrate, apicidin) have been shown to increase global K_L‐la_ of H3 [40].

Despite considerable advances, several critical challenges remain unresolved. First, the issue of off‐target effects presents a significant barrier: p300 inhibitors can lead to broad alterations in acetylation patterns, while modulators of HDACs often influence multiple post‐translational modifications beyond acetylation. Furthermore, achieving tissue specificity remains difficult. Systemic inhibition of AARS1 or AARS2, for example, may inadvertently disrupt essential alanine metabolism in normal tissues. Finally, the dual functional roles of certain targets complicate therapeutic strategies. Although activation of SIRT3 has been shown to reduce levels of CCNE2 lactylation, exerting anti‐tumour effects, it may simultaneously impair mitochondrial adaptive responses, highlighting a potential trade‐off in its pharmacological activation.

#### 1.9.4 Targeting Lactylation Sites

**FIGURE 9:**
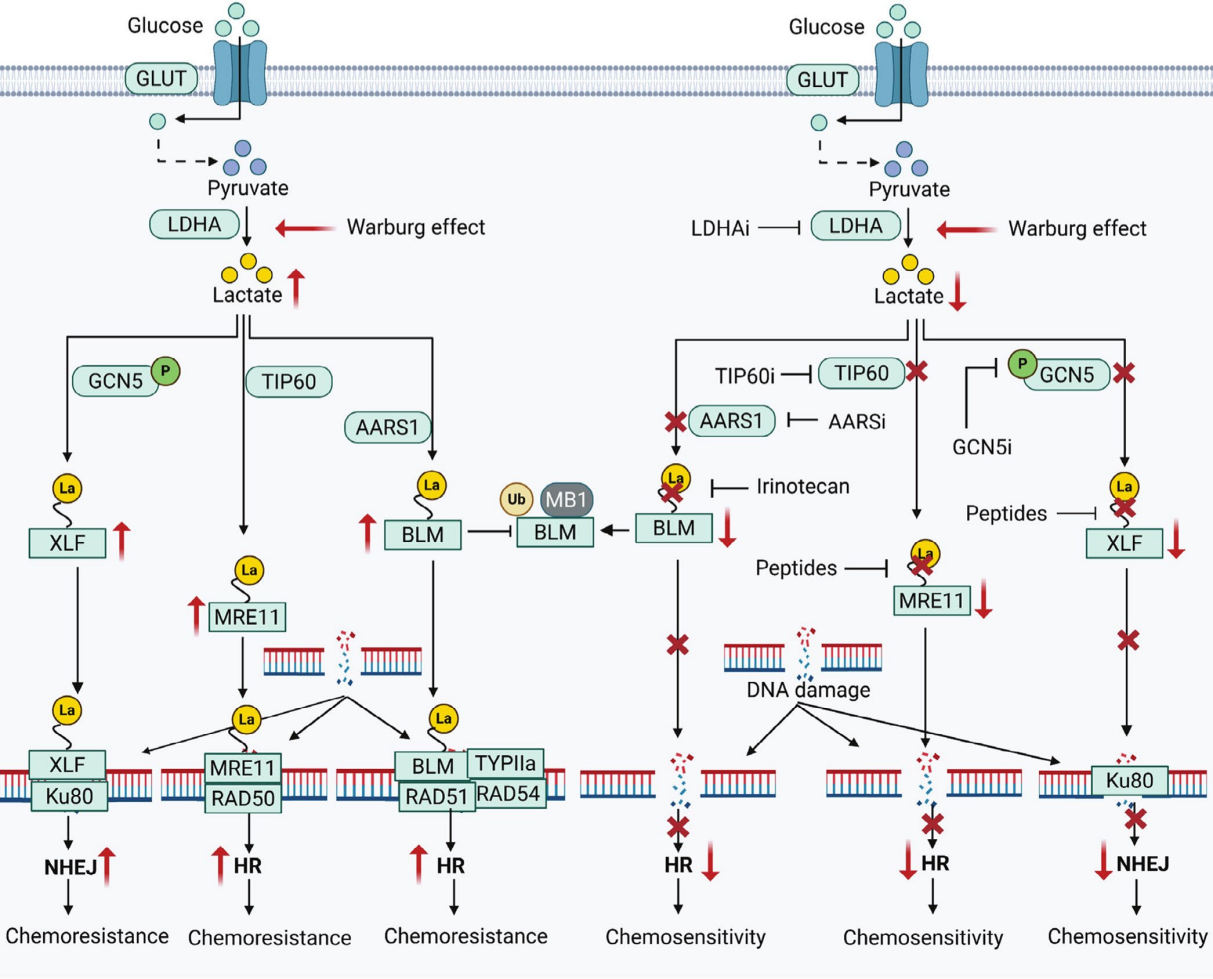
The lactylation–DNA repair axis in chemoresistance and its therapeutic targeting via direct modulation of lactylated substrates. The left panel illustrates how glycolytic reprogramming induces lactylation of DNA repair proteins (XLF, MRE11, BLM), consequently HR and NHEJ and leading to chemoresistance. Conversely, the right panel outlines therapeutic strategies that directly target lactylated substrates modulating this axis. Notably, inhibition of BLM lactylation in combination with irinotecan overcomes anthracycline resistance in preclinical models and has shown safety and feasibility in a phase I clinical trial.

Targeting lactylation sites on substrates directly represents a viable therapeutic approach (Figure 9).

Anthracyclines such as epirubicin (EPI) confer antitumor activity mainly through DNA intercalation and induction of DNA damage [128, 240]. However, K_L‐la_ of the HR helicase BLM confers resistance to EPI by enhancing HR‐mediated DNA repair in bladder cancer cells. Preclinical data show that the small molecule irinotecan suppresses BLM lactylation, impairs HR activity and restores anthracycline sensitivity [3]. A single‐arm phase I clinical trial (NCT06766266) shows that irinotecan liposomes combined with EPI is a safe and feasible approach for patients with anthracycline‐resistant recurrent bladder cancer [3]. Likewise, in CRC, XLF‐K288la facilitates non‐homologous end joining (NHEJ) repair and an L‐lactylation‐specific peptide inhibitor targeting XLF‐K288 impairs XLF–Ku interactions, disrupts NHEJ, and synergizes with 5‐fluorouracil to suppress tumorigenesis in patient‐derived xenograft models [43]. MRE11 lactylation activates HR to confer chemoresistance and inhibition of this modification with a peptidic inhibitor (K673‐peptide‐3#) promotes chemotherapy sensitivity [67]. In addition to DNA repair, oncogenic signalling and transcriptional programs are similarly induced by substrate‐specific K_L‐la_. A small‐molecule drug screen demonstrated that tubuloside A inhibits ABCF1‐K430la and suppresses HCC progression [163]. Similarly, a cell‐penetrating peptide specifically inhibiting K_L‐la_ of ERK impairs tumorigenesis in KRAS‐mutant tumour models [24].

Yet, site‐specific modulation of K_L‐la_ seems a promising strategy for precision drug development. However, its translation into clinical applications will depend on a deeper understanding of the underlying molecular mechanisms and careful assessment of therapeutic efficacy, safety and tolerability.

## 2 Conclusion and Perspective

L‐Lactate has transcended its historical perception as a metabolic waste product, emerging as a critical regulator of cellular processes through K_L‐la_. While significant progress has been made in characterising K_L‐la_ enzymes, including classical acyltransferases (KATs) and the recently discovered AARS1/2, key questions remain regarding their substrate specificity, regulatory mechanisms and functional consequences. Future research must elucidate how these enzymes select their targets and how K_L‐la_ integrates with other post‐translational modifications to influence protein localisation, stability and function. A critical gap lies in identifying dedicated ‘reader’ proteins that interpret K_L‐la_ marks, analogous to those recognising acetylation or methylation, which will be essential for deciphering lactylation's broader biological impact. Additionally, given that K_L‐la_, K_ce_ and K_d‐la_ are structurally and mechanistically distinct PTMs [45], rigorous stereochemical annotation is indispensable. During investigation, explicit specification of the lysine enantiomer and of the lactyl donor (L‐ or d‐lactate/lactyl‐CoA) is required to avoid misinterpretation of lactylation pathways and to ensure experimental reproducibility.

To better understand the physiological functions of K_L‐la_, it is crucial to perform comprehensive quantification in physiological and pathological contexts. Technological advances, such as CRISPR‐Cas9‐mediated targeting of lactyltransferases/delactylases and site‐specific K_L‐la_ modulation, will enable precise functional dissection in physiology and disease. Notably, the involvement of K_L‐la_ in glycolysis‐dependent processes, spanning cancer, immunity, neurodegenerative disorders and cardiovascular disease, highlights its therapeutic potential. Promisingly, targeted interventions demonstrate the feasibility of L‐lactylation‐directed therapies. For example, a peptide inhibitor combined with 5‐fluorouracil suppressed XLF‐lactylation, thereby blocking NHEJ and enhancing chemosensitivity [43]. Similarly, synergistic irinotecan treatment inhibits the K_L‐la_ of BLM and effectively reversed anthracycline resistance in preclinical models, while a phase I clinical trial demonstrated the safety and feasibility of this combination therapy [3]. Strategic combination approaches, integrating K_L‐la_ modulation with chemotherapy, immunotherapy or cell therapy, may enhance treatment efficacy while minimizing off‐target effects.

Although the preclinical results are encouraging, several challenges remain in clinical translation. First, a major concern is targeting selectivity. Many currently available compounds, including p300 inhibitors and HDAC modulators, influence multiple PTMs such as acetylation in addition to lactylation, making it difficult to attribute therapeutic benefits to K_L‐la_ inhibition. Similarly, metabolic interventions targeting LDHA, PKM2 or lactate transporters may broadly alter cellular metabolism and disrupt physiological processes in normal tissues. Second, despite encouraging efficacy in experimental models, evidence supporting durable in vivo target engagement, pharmacological selectivity and long‐term safety remains limited. Addressing these challenges will require the development of substrate‐specific therapeutic strategies, robust approaches for monitoring lactylation dynamics in vivo, and delivery platforms capable of restricting drug activity to disease‐relevant tissues.

Moreover, although the role of several lactylated proteins including BLM and XLF in DNA repair and chemoresistance has been elucidated [3, 43], the functions of other differentially lactylated proteins are largely unknown. Most importantly, K_L‐la_‐induced protein structural changes are worth examining in future research. Moving forward, systematic quantification of K_L‐la_ across physiological and pathological contexts, together with mechanistic dissection, development of selective pharmacologic tools and translation of these insights into clinically viable strategies will be essential to harness K_L‐la_'s full therapeutic potential and solidify its role as a cornerstone of metabolic‐epigenetic regulation.

## Author Contributions

**Anoosha Malik, Muhammad Dilawar, Junguang Liao, Qitao Qian, Ming Xu, Jie Zheng, Sisi Lin, Xiaobo Zhu:** data analysis, literature formation. **Anoosha Malik, Muhammad Dilawar, Junguang Liao, Qitao Qian, Ming Xu, Xiaobo Zhu:** validation and revision. **Guiqian Chen, Limin Jin, Qiuwei Ge:** literature formation, supervision and grant holder.

## Funding

This work was supported by grants by National Natural Science Foundation of China (82571024, 81400489), Zhejiang Provincial Natural Science Foundation of China (LZ23H140001) and the Fundamental Research Funds of Zhejiang Sci‐Tech University (26042139‐Y).

## Ethics Statement

The authors have nothing to report.

## Consent

All the authors agree to the current state of the manuscript to be submitted to the journal.

## Conflicts of Interest

The authors declare no conflicts of interest.

## Data Availability

The data that support the findings of this study are available in this article.
